# Effect of Long-Term Farming Practices on Agricultural Soil Microbiome Members Represented by Metagenomically Assembled Genomes (MAGs) and Their Predicted Plant-Beneficial Genes

**DOI:** 10.3390/genes10060424

**Published:** 2019-06-03

**Authors:** Johanna Nelkner, Christian Henke, Timo Wentong Lin, Wiebke Pätzold, Julia Hassa, Sebastian Jaenicke, Rita Grosch, Alfred Pühler, Alexander Sczyrba, Andreas Schlüter

**Affiliations:** 1 Center for Biotechnology (CeBiTec), Bielefeld University, Genome Research of Industrial Microorganisms, Universitätsstraße 27, 33615 Bielefeld, Germany; jnelkner@cebitec.uni-bielefeld.de (J.N.); tlin@cebitec.uni-bielefeld.de (T.W.L.); jhassa@cebitec.uni-bielefeld.de (J.H.); puehler@cebitec.uni-bielefeld.de (A.P.); 2 Center for Biotechnology (CeBiTec), Bielefeld University, Computational Metagenomics Group, Universitätsstraße 27, 33615 Bielefeld, Germany; christian@cebitec.uni-bielefeld.de (C.H.); WiebkePaetzold@gmx.de (W.P.); asczyrba@cebitec.uni-bielefeld.de (A.S.); 3 Justus-Liebig-University Gießen, Bioinformatics & Systems Biology, Heinrich-Buff-Ring 58, 35392 Gießen, Germany; Sebastian.Jaenicke@computational.bio.uni-giessen.de; 4 Leibniz-Institute of Vegetable and Ornamental Crops (IGZ) Großbeeren/Erfurt eV, Theodor-Echtermeyer-Weg 1, 14979 Großbeeren, Germany; Grosch@igzev.de

**Keywords:** soil microbiome, suppressive soil, biocontrol, plant–growth–promotion (PGP), metagenomic binning, metagenomically-assembled-genomes (MAGs), secondary metabolite synthesis, carbon dioxide fixation, carbohydrate-active enzymes, differentially abundant features (DAFs)

## Abstract

To follow the hypothesis that agricultural management practices affect structure and function of the soil microbiome regarding soil health and plant-beneficial traits, high-throughput (HT) metagenome analyses were performed on Chernozem soil samples from a long-term field experiment designated LTE-1 carried out at Bernburg-Strenzfeld (Saxony-Anhalt, Germany). Metagenomic DNA was extracted from soil samples representing the following treatments: (i) plough tillage with standard nitrogen fertilization and use of fungicides and growth regulators, (ii) plough tillage with reduced nitrogen fertilization (50%), (iii) cultivator tillage with standard nitrogen fertilization and use of fungicides and growth regulators, and (iv) cultivator tillage with reduced nitrogen fertilization (50%). Bulk soil (BS), as well as root-affected soil (RS), were considered for all treatments in replicates. HT-sequencing of metagenomic DNA yielded approx. 100 Giga bases (Gb) of sequence information. Taxonomic profiling of soil communities revealed the presence of 70 phyla, whereby *Proteobacteria*, *Actinobacteria*, *Bacteroidetes*, *Planctomycetes*, *Acidobacteria*, *Thaumarchaeota*, *Firmicutes*, *Verrucomicrobia* and *Chloroflexi* feature abundances of more than 1%. Functional microbiome profiling uncovered, i.a., numerous potential plant-beneficial, plant-growth-promoting and biocontrol traits predicted to be involved in nutrient provision, phytohormone synthesis, antagonism against pathogens and signal molecule synthesis relevant in microbe–plant interaction. Neither taxonomic nor functional microbiome profiling based on single-read analyses revealed pronounced differences regarding the farming practices applied. Soil metagenome sequences were assembled and taxonomically binned. The ten most reliable and abundant Metagenomically Assembled Genomes (MAGs) were taxonomically classified and metabolically reconstructed. Importance of the phylum *Thaumarchaeota* for the analyzed microbiome is corroborated by the fact that the four corresponding MAGs were predicted to oxidize ammonia (nitrification), thus contributing to the cycling of nitrogen, and in addition are most probably able to fix carbon dioxide. Moreover, *Thaumarchaeota* and several bacterial MAGs also possess genes with predicted functions in plant–growth–promotion. Abundances of certain MAGs (species resolution level) responded to the tillage practice, whereas the factors compartment (BS vs. RS) and nitrogen fertilization only marginally shaped MAG abundance profiles. Hence, soil management regimes promoting plant-beneficial microbiome members are very likely advantageous for the respective agrosystem, its health and carbon sequestration and accordingly may enhance plant productivity. Since Chernozem soils are highly fertile, corresponding microbiome data represent a valuable reference resource for agronomy in general.

## 1. Introduction

Soil is of fundamental importance for food production since it provides resources enabling crop cultivation. The European Commission considers soil as a non-renewable resource, since the process of soil formation is extremely slow [1,2]. Among the soil biota, bacteria, archaea, algae and fungi contribute to soil functions, especially biological soil activity and fertility. In ‘healthy’ soils, the majority of microorganisms are assumed to be beneficial or neutral for plant growth. However, soil microbiome members may also feature detrimental properties such as pathogenic effects on plants. In intensive farming, protection of plants against phytopathogens often involves application of pesticides. Several negative effects have been attributed to pesticide usage, such as impairments of soil functions, often accompanied by crop yield losses in the long term [3]. Intensive farming can also cause accumulation of plant pathogens in soils because of short crop rotation cycles. Moreover, application of agrochemicals and pesticides may also have negative effects on beneficial soil microbiota and cause development of resistances in phytopathogens [4,5]. To preserve agricultural soils for future food supply, more efficient and sustainable farming strategies have to be developed. Currently, little is known about how farming practices such as tillage techniques and nitrogen fertilization intensities affect soil microbiomes. It is supposed that the composition of a soil community is linked to its ability to suppress plant pathogens [6]. A better understanding of these interrelationships is needed to improve agricultural farming strategies and to achieve intended sustainability.

Recent studies have shown that soil microbial communities differ considerably with respect to cultivated plants, soil parameters and treatments [6,7,8,9]. Soil amendments like green manure, dung and chitin affect soil microbiome compositions and soil suppressiveness [10,11,12,13]. It has been hypothesized that disease suppressiveness is mediated by microbial consortia composed of multiple species rather than single microorganisms [14]. However, only few recent studies focused on the influence of long-term farming practices involving different tillage techniques and nitrogen fertilization intensities on soil characteristics comprising taxonomic and functional profiles of microbial soil communities [15,16,17]. Previous studies addressing taxonomic community profiling showed that nitrogen fertilization is a main driver of bacterial community development [18]. Indicators of a ‘healthy’ or disease suppressive soil microbiome are i.a. presence of biocontrol species such as those belonging to the fungal genera *Mortierella*, *Trichoderma*, *Fusarium*, and *Malassezia*, or to the bacterial genera *Pseudomonas*, *Bacillus*, and *Burkholderia* that are often abundant in soils featuring general disease suppressiveness [19]. Non-targeted metagenomics, the direct sequencing of metagenomic DNA, has been proposed to be the most accurate approach for the assessment of the impact of farming practices on the soil microbiome composition [20,21,22]. Non-targeted metagenomics in contrast to marker-gene-targeted metagenomics has the advantage that microbiome diversity and functions can be analyzed in parallel. Whole metagenome shotgun data can be classified directly in read-based approaches, which often is demanding due to the short read lengths of most high-throughput sequencing technologies. Moreover, short single sequences do not provide genetic context information. Alternatively, deeply sequenced metagenomes can be assembled and binned to possibly reconstruct single genomes. Since both approaches have advantages and limitations, it has been proposed to use both strategies to get the most out of metagenome sequence datasets [22]. Taxonomic community profiling preferably is done directly on metagenomic single read sequences, since an almost unbiased view into the composition of the underlying microbiome can be achieved. On the other hand, metagenome assembly usually reduces the amount of data to be analyzed by magnitudes. Analysis of assembled genes and their encoded gene products provides insights into functional characteristics of the community and discloses unique metabolic capabilities associated with a particular environment [23]. Challenges in the assembly-based approach refer to the reconstruction of closely related genomes on the strain or species level. Ideally, metagenomically assembled genomes (MAGs), compiled by application of assembly and binning tools, offer the opportunity to analyze genetic information in its genomic context.

In this study, whole metagenome shotgun sequencing was applied to comparatively analyze the impact of different long-term farming practices involving tillage techniques (plough vs. cultivator tillage) and nitrogen (N) fertilization intensities (intensive N-fertilization including pesticide application vs. extensive N-fertilization without fungicide use) on the microbial community structure and function of agricultural soils. It was hypothesized that extensive farming is more sustainable due to promotion of plant-beneficial microorganisms. Moreover, it was assumed that soil communities in extensively managed soils encode traits related to suppressiveness against phytopathogens. Taxonomic and functional profiling of the soil microbiomes under different farming practices analyzed in this study were initially done on a single read basis. To achieve a resolution at the gene level, metagenome assemblies were calculated. Taxonomic contig binning approaches led to the compilation of metagenomically assembled genomes (MAGs) representing dominant soil community members. Several of the compiled MAGs represent putative plant-growth-promoting, plant-beneficial and/or carbon- and nitrogen-cycling species. Differentially abundant MAGs in response to farming practices were identified.

## 2. Materials and Methods

### 2.1. Analyzed Soil Samples and Extraction of Total Microbial DNA

Soil samples were obtained from the long-term experiment (LTE-1) located in Bernburg, Germany (51.82° N, 11.70° E). Corresponding field plots were established by the Anhalt University of Applied Sciences in 1992. The LTE-1 soil was classified as a loess chernozem over limestone (8% sand, 70% silt, 22% clay). In this study, the effect of different farming practices on the soil microbiome was analyzed. The microbiome in soil under conservative cultivator tillage (CT, 10 cm depth, non-inverting, soil loosening) was compared to those under conventional tillage (P, mould-board plough, ploughing depth 30 cm, including soil inversion). In addition, the differently managed soils were either under intensive (Int) nitrogen (N) supply and common pesticide application or under extensive (Ext) N-fertilization without fungicides and growth regulator use. Hence, the study comprised the following treatments: P-Int vs. CT-Int and P-Ext vs. CT-Ext [24]. Soil sampling was done after harvest of winter wheat (*Triticum aestivum*) at the depth of 0 to 30 cm in 2015. Metadata of the LTE-1 field site are summarized in Table 1. Samples were taken in a spatial randomly distributed scheme as combined samples of 15 soil cores each. Soil samples were naturally dried outside, sieved (4 mm mesh) and stored in the dark at 6 °C until further processing. The soil microbiome was analyzed from bulk soil (BS) and from soil affected by the roots of the model plant lettuce (*Lactuca sativa* var. capitata cv. ‘Tizian’, Syngenta, Bad Salzuflen, Germany). For this purpose, growth chamber experiments were performed. Initially, soils were incubated for two weeks in the dark (20 °C day/15 °C night, 60% RH/80% RH) with 100 hPa water potential (T5 tensiometer, UMS AG, Munich, Germany). Afterwards, lettuce was sown in the respective soils from the LTE field plots for germination and the seedlings were cultivated until they reached the 3 to 4 leaf stage before transfer of individual plants into pots (10 × 10 × 11 cm) containing the respective soils. To ensure that each plant in each treatment had comparable amounts of nitrogen available, the N contents of soils were measured before planting. Soils were fertilized using calcium nitrate to 50% of the recommended amounts of N for lettuce growth (0.32 g/pot) during planting of seedlings and the remaining amount was added after two weeks. Each treatment comprised four replicates with four plants per replicate (repetitions) arranged in randomized block design in the growth chamber, and four replicates of bulk soil (BS). BS samples were transferred to separate pots without lettuce plants. Lettuce plants were cultivated at 20 °C day/15 °C night, 60% RH/80% RH, 16 h day with 420 μmol m^−2^ s^−1^ photosynthetic active radiation and 100 hPa water potential. The plants were harvested after ten weeks. Root affected soil samples (RS) were taken from pots with lettuce plants at harvest. Per replicate, soil was pooled from two out of the four plant experiment repetitions, prior to DNA extraction. Total microbial community (TC)-DNA was extracted from 500 mg soil per sample using the FastDNA™ SPIN Kit for Soil (MP Biomedicals, Hamburg, Germany).

### 2.2. Metagenome Library Preparation, Sequencing and Preprocessing of Sequencing Data

Per treatment (P-Int-BS, CT-Int-BS, P-Ext-BS, CT-Ext-BS; P-Int-RS, CT-Int-RS, P-Ext-RS, CT-Ext-RS), two replicates (consisting of two repetitions each) were selected arbitrarily for sequencing. Extracted TC-DNA was processed to yield 16 Illumina TruSeq sequencing libraries for HT-sequencing on the Illumina HiSeq system (2 × 250 bp paired-end reads, HiSeq Rapid SBS kit v2 (500c), two lanes per sample). Metagenome sequencing yielded between 45 Mio and 69 Mio raw sequence reads (in total 505 Mio. reads) for each treatment regime (in replicates). Obtained reads were trimmed using Trimmomatic [26] version 0.36 using adapter template TruSeq3-PE and a minimum length of 30 bp. Remaining PhiX matching reads were filtered using BBDuk (v 36.84, Bushnell, http://jgi.doe.gov/data-and-tools/bbtools/). During adapter and quality trimming, 5.17 to 15.9% of raw sequences had to be discarded. The resulting high-quality reads were subjected to read-based analyses as well as assembly-based analyses.

### 2.3. Taxonomic and Functional Microbiome Analyses Based on Single Read Data

For read-based analysis, the reads were uploaded into the MGX platform [27]. The therein implemented pipelines for taxonomic classification (MGX taxonomic classification, based on Kraken and Diamond vs. RefSeq proteins), Enzyme Commission (EC) number annotation (best-blast-hit vs. SwissProt database) and Clusters of Orthologous Groups proteins (COG)-based functional classification were applied with default configuration (FilterBlastHits 1e-5 cutoff, 80% relative identity).

### 2.4. Metagenome Assembly and Binning

The preprocessed reads were assembled using MEGAHIT (v 1.1.1; preset: meta-large) [28]. Assembled contigs greater than 1 kb were further subjected to structural annotation using MetaProdigal (v 2.6.3) [29]. The predicted coding sequences then were functionally annotated using DIAMOND (v 0.8.36) [30] against the databases National Center for Biotechnology Information non-redundant protein sequences database (NCBI-nr) (cutoff 0.1), Pfam and KEGG (cutoff 1e-5). Using MEGAN6 Community Edition [31], the genes were taxonomically classified by means of the lowest common ancestor (LCA) algorithm. Reads were mapped back onto the assembly using BBMap (v 36.84, Bushnell, http://jgi.doe.gov/data-and-tools/bbtools/). The assembled contigs were binned using MetaBat (v 2.12.1) [32] and MaxBin (v 2.0) [33] and, subsequently, metagenomically assembled genomes (MAGs) were constructed by integrating the binning results applying DAS Tool [34]. For exploration of calculated observations and in order to inspect functional annotations and binning results, assembled genes, contigs and bins were imported into the Elastic MetaGenome Browser (EMGB) platform [35]. EMGB is a fast web-based viewer for metagenomic analyses featuring various visualizations, filtering options and comparisons. LCA of MAGs was determined by the LCA assignment based on genes. If more than 50% of all genes in a binned genome were assigned to this taxon, we assigned the complete binned genome to this taxon. Furthermore, MAGs were classified according to the Genome Taxonomy Database [36] using GTDB-Tk (https://github.com/Ecogenomics/GtdbTk).

### 2.5. Functional Analyses of Obtained Metagenomically Assembled Genomes (MAGs)

MAGs were annotated in RAST [37] using the default pipeline (RASTtk), selecting the appropriate domain. File formats needed for the downstream analysis were downloaded from the RAST server. Carbohydrate-active enzymes (CAZymes) were annotated by means of dbCAN2 (v7.0) [38], with predicted protein sequences as input file and all options activated, including the CGCFinder option activated in order to predict CAZyme gene clusters (CGCs). Hits were counted if they were predicted by more than two methods. In silico screening for secondary metabolite biosynthetic gene clusters was conducted with antiSMASH version 4.2.0 [39,40] with fasta files as input and the option ‘all extra features on’. Metabolic pathways were predicted using the Kyoto Encyclopedia of Genes and Genomes (KEGG) tools BlastKOALA (v 2.1) or GhostKOALA (v 2.0) [41], if the MAG to be analyzed comprised more than 5000 predicted genes.

### 2.6. Detection of Differentially Abundant Features (DAFs)

Abundance and biostatistical analyses were performed within Calypso version 8.84 [42] including 162 MAGs (>20% completeness and ≤15% contamination by means of CheckM). Reads Per Kilobase per Million (RPKM) abundance tables of MAGs were used as input for Calypso, selecting the ‘Calypso OTU table with tax’ format. For relative abundance data of genera from direct classification of reads exported from MGX, the same workflow was applied. Filtering and normalization were turned off. Samples were arranged to blocks by factor tillage, fertilization intensity and compartment in order to identify the features affected by the single factors. Considering the small sample size of 162 MAGs, the cut-off logarithmic LDA score in the LEfSe analysis was set to 3.0 [43].

### 2.7. Sequence Submission to a Public Sequence Archive

Metagenome sequence data, assembled contigs and MAGs were submitted to the European Nucleotide Archive (ENA) and are archived under the study accession number PRJEB31111.

## 3. Results and Discussion

### 3.1. Layout and Soil Cultivation Treatment Regime at the Long-Term Experimental Field Site (LTE-1) Located at Bernburg-Strenzfeld (Saxony-Anhalt, Germany)

**Figure 1:**
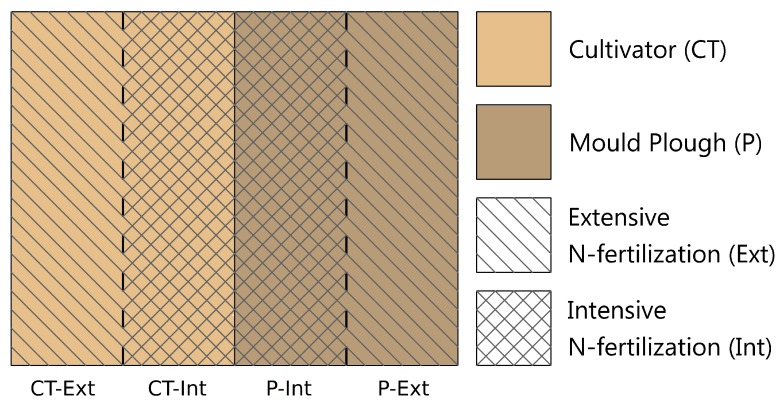
Schematic representation of the soil cultivation treatment regime of the studied parcel of the LTE-1 (long-term experimental) field site at BBG-Strenzfeld (Bernburg, Saxony-Anhalt). Field strips were treated as follows: (i) ploughed with standard (intensive) N-fertilization and application of fungicides and growth regulators (P-Int), (ii) ploughed with reduced N-fertilization (50% of intensive, P-Ext), (iii) cultivator treatment with standard N-fertilization and application of fungicides and growth regulators (CT-Int) and (iv) cultivator treatment with reduced fertilization (CT-Ext). Subsequently to a greenhouse experiment involving lettuce as as model plant, total community (TC)-DNA was extracted considering the additional factors bulk soil (BS) and root-affected soil (RS).

To follow the hypothesis that soil management practices affect structure and function of the soil microbiome regarding soil health and enrichment of plant-beneficial community members, the metagenome of the LTE-1 (Long-Term-Experiment 1) field site at Bernburg-Strenzfeld (Saxony-Anhalt, Germany) was analyzed by high-throughput (HT) metagenome sequencing. Field plots and the cultivation treatment scheme of the LTE-1 site are shown in Figure 1. Soil metagenomic DNA was extracted and sequenced in replicates for four management practices (as illustrated in Figure 1) considering bulk soil (BS) and root-affected soil (RS). Treatments comprised mould-board ploughing (P) vs. cultivator treatment (CT, reduced tillage) in combination with intensive (Int) vs. extensive (Ext) nitrogen fertilization. Intensive fertilization was combined with fungicide and growth regulator application and ‘extensive nitrogen fertilization’ refers to a reduction of fertilization by 50%, as described previously [24]. Recently, the LTE-1 field site analyzed in this study has been described in detail including information on its soil type (a loess chernozem soil over limestone), soil parameters, soil management history, crop yields and additional metadata [24]. Sampling at the LTE-1 field site for microbiome metagenome sequencing on the Illumina HiSeq system was done in parallel to the previously published study on fungal community profiling [24], namely in July 2015. The statistics of obtained high-quality reads per dataset is given in Table S4. In total, almost 100 Gb of sequence information (ca. 440 Mio. high-quality reads) was obtained from 16 metagenomic datasets (4 treatments for bulk soil and root-affected soil in two replicates, see Table S4).

### 3.2. Unraveling the Soil Microbial Community Composition and Functional Potential of the LTE-1 Field Site Based on Metagenome Single Read Analyses

**Figure 2:**
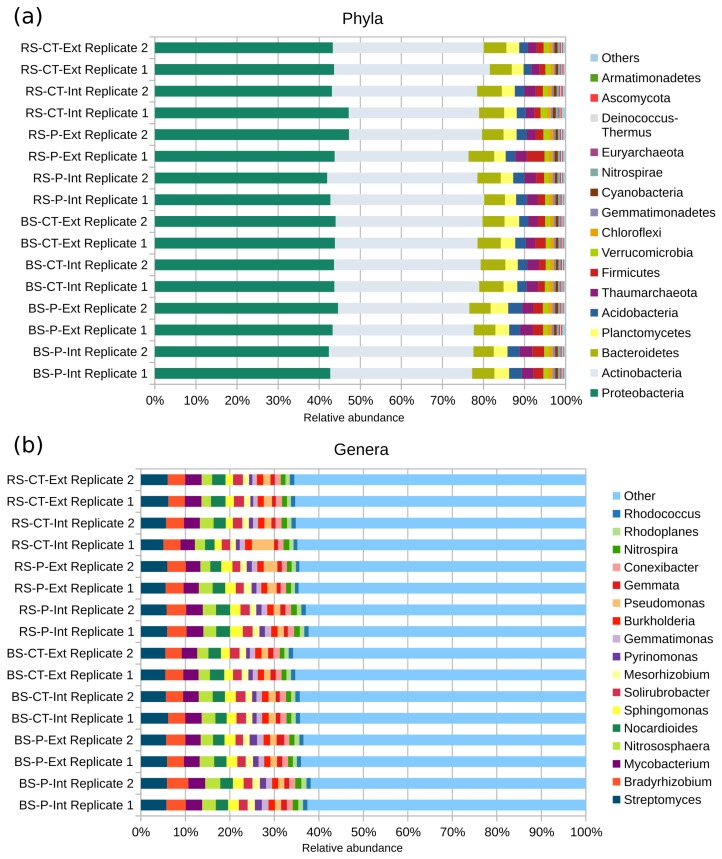
Taxonomic profiling of the soil microbial communities residing in the analyzed LTE-1 field plots on the rank of (**a**) phyla and (**b**) genera. Relative abundances of the most abundant phyla/genera are shown and were determined based on direct classification of reads normalized to the dataset sizes (fractions). In addition, 54 phyla and 3075 genera with relative abundances of less than one per mill or one percent, respectively are compiled as ‘Others’. The colors are ordered right-to-left according to the legend top–down. Abbreviations stand for bulk soil (BS) or root-affected soil (RS), tillage by mould-board plough (P) or cultivator (CT), and nitrogen fertilization intensive (Int) or extensive (Ext).

To deduce the taxonomic profiles of the soil microbiomes analyzed, the bioinformatics tool Kraken for metagenomic sequence classification was applied [44]. In total, 36.25% of all reads could be classified, identifying 70 phyla. The following phyla with descending relative abundances of more than one per mill were identified: *Proteobacteria*, *Actinobacteria*, *Bacteroidetes*, *Planctomycetes*, *Acidobacteria*, *Thaumarchaeota*, *Firmicutes*, *Verrucomicrobia*, *Chloroflexi*, *Gemmatimonadetes*, *Cyanobacteria*, *Nitrospirae*, *Euryarchaeota*, *Deinococcus-Thermus*, *Ascomycota* and *Armatimonadetes* (see Figure 2). On genus rank, 3092 different genera were identified. The most abundant genera are depicted in Figure 2 and include *Streptomyces* with relative abundances of 5.0–6.2%, followed by *Bradyrhizobium* (3.7–4.8%), *Mycobacterium* (3.0–3.8%), *Nitrososphaera* (2.1–3.4%), and *Nocardioides* (2.1–3.3%). Classifications at other taxonomic ranks are provided in the Supplementary Material of this article (Figures S1–S5). Overall, the composition of the LTE-1 microbiome is in accordance with microbiomes from other agricultural soil environments [45,46,47]. Pronounced differences in microbiome compositions were not observed on higher taxonomic ranks when comparing samples that underwent different soil treatment practices. The obtained taxonomic profiles were searched for genera comprising putative plant-growth-promoting (PGP) and soil-health-ameliorating species that are listed in Figure S6. Many *Pseudomonas* species identified in the LTE-1 microbiome are known to encode plant-beneficial traits [48,49]. Further genera of interest detected include *Burkholderia*, *Arthrobacter*, *Bacillus*, *Azospirillum*, *Azotobacter* and *Trichoderma* [19,49,50,51,52,53]. This study presents the first metagenome-based taxonomic profiling for the microbiome of a loess chernozem soil located in the central part of Germany (Magdeburger Börde). This soil type is characterized by its outstanding fertility [54] and therefore corresponding microbiome data represent a valuable reference resource for studying similar agricultural soils.

**Figure 3:**
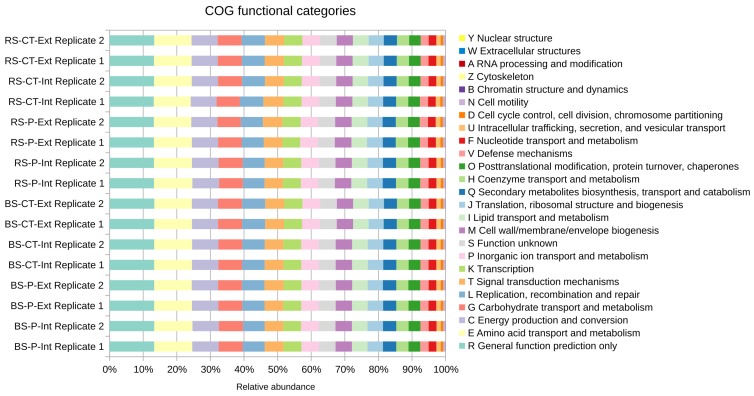
Functional profiling on the level of Clusters of Orthologous Groups of proteins (COG) functional categories of the microbial communities residing in the differently treated LTE-1 soils based on direct classification of reads. Relative abundances were determined based on direct classification of reads normalized to the dataset sizes (fractions). Colors are ordered right-to-left according to the legend top-down. Abbreviations stand for bulk soil (BS) or root-affected soil (RS), tillage by mould-board plough (P) or cultivator (CT), and nitrogen fertilization intensive (Int) or extensive (Ext).

Functional assignments to COG categories revealed that for most reads only a general functional prediction can be made (category R), indicating that many sequences of the microbiome represent still unknown functions (see Figure 3). The category ‘Amino acid transport and metabolism’ (E) is on rank two suggesting that amino acid metabolism is of prominent importance for the soil microbiome analyzed. Amino acids are abundant metabolites in plant root exudates and are supposed to shape structures of rhizosphere microbiomes since they serve as important nutrients of particular community members. The dominant COG categories E, C (Energy production and conversion) and R were recently also reported for soil samples from China (Sichuan province; [13]) suggesting general (global) relevance of corresponding functions for soil microbiomes. However, no remarkable differences were observed for the main COG categories regarding soil treatment practices applied at the LTE-1 field plots of this study.

At the level of COG numbers (Figure S11), abundant assignments mostly refer to basic housekeeping functions such as dehydrogenases (COG1028, COG1960, COG673, COG277), hydrolases and acetyl-transferases (COG596), glycosyl-transferases (COG438, COG463) and response regulators (COG2204, COG2197, COG745). The most dominant COG number 642 (signal transduction histidine kinase) indicates the importance of signaling pathways and COG577 (ATP-binding cassette (ABC)-type antimicrobial peptide transport) points towards defense mechanisms facilitating enhanced competitiveness of corresponding host microorganisms in the soil environment. Interestingly, non-ribosomal peptide synthetase (NRPS) modules and related protein functions (COG1020) are among the top 25 most abundant COG assignments and are slightly enriched in samples that underwent mould-board plough tillage (P). However, abundant COG numbers do not vary profoundly between soil treatment practices. A similar picture appeared with respect to functional profiling based on EC (Enzyme Commission) numbers (see Figure S7).

### 3.3. Assembly of Metagenomic Reads Yielded Approx. 6.9 Mio. Genes Characterizing the LTE-1 Microbiome

Due to short read lengths, metagenome classification based on single read sequences has the limitation that differentiation of functional genes originating from different species is difficult and often impossible. As a matter of course, genetic context information also is not available from single-read analyses. Accordingly, there is a trend towards *de novo* assembly- and binning-based metagenome analyses [55] which also are practicable for the soil metagenome datasets of this study since they were deeply sequenced (approx. 100 Gb total sequence information). Secondly, progress in the development of advanced bioinformatics assembly and binning tools facilitate genome-centered analyses of complex metagenomes [32,33,56,57,58,59].

A combined assembly of all 16 metagenome sequence datasets obtained for the LTE-1 field-site by means of the MEGAHIT assembler [28] yielded 3,012,901 contigs featuring a minimal length of 1000 bp and a maximal length of 206 kb. The total assembly size amounted to 4.9 Gb. On average, 48% of input reads mapped back to the assembly. Indicator values of the assembly statistics are summarized in Table S5. The large proportion of small contigs may result from assembly problems due to the high diversity of the soil community as already shown by single read analyses. Secondly, very similar sequences originating from closely related species or strains of species previously were shown to impede assemblies [60]. Gene prediction, applying MetaProdigal [29], resulted in identification of 6,902,107 presumptive genes featuring a summed-up total length of 4.4 Gb. Approximately one third of the predicted genes appeared to comprise complete coding sequences (CDSs). Hence, with this approach, a resolution at the gene level was achieved and longer contigs enable evaluation of the genetic context for genes of interest meaning consideration of corresponding operon structures and/or elucidation of functional gene clusters. Most of the deduced gene products (85.85%) produced a hit against the NCBI nr protein sequence database (see Table S5) and/or the protein families database Pfam [61] and/or the KEGG (Kyoto Encyclopedia of Genes and Genomes) database [62].

Mapping of gene products encoded by assembled genes to KEGG pathways revealed that the maps representing ABC transporters, purine and pyrimidine metabolism, two-component systems, carbon fixation, oxidative phosphorylation, amino-acid metabolism (arginine, proline, glycine, serine, and threonine), pyruvate and methane metabolism received the most assignments (see Supplemental Table S2). ABC-transporters are of importance for import of available substrates such as inorganic and organic ions, mono- and oligosaccharides, amino-acids and peptides. Numerous two-component-system assignments illustrate the basic necessity for soil community members to respond to changing environmental conditions for rapid adaptation purposes. Amino acids are major compounds in plant root exudates and therefore their re-utilization by soil microorganisms is an inevitable implication explaining the importance of enzymes functioning in amino-acid metabolism.

Since the importance of plant-growth-promoting (PGP) microbiome members for plant productivity is a central concern of this article, the set of assembled genes was examined for the occurrence of putative determinants predicted to contribute to plant–growth–promotion. PGP marker genes have recently been compiled in a review article [63] and therefore guided the analysis that was done here. Strikingly, a very large number of genes were assigned to the biochemical reactions and pathways involving organic or inorganic nitrogen compounds such as nitrogen fixation, nitrification, denitrification, assimilatory nitrate reduction, anaerobic ammonium oxidation (anammox) and the interconversion of nitrogenous organic compounds (see Table S3). Nitrogen is a limiting macronutrient regarding plant growth. Therefore, enzymes catalyzing synthesis of nitrogen compounds usable by plants are of importance. Among the assembled nitrogen metabolism genes, denitrification genes also comprising *nirK* were identified. NirK encodes a subunit of the nitric oxide-forming nitrite reductase and is of importance since NO (nitric oxide) is involved in signaling to plants ultimately inducing alterations in plant root growth in an auxin-dependent manner [64]. Microorganisms and plants also compete for phosphorus. Therefore, solubilisation of mineral phosphorus by microorganisms contributes to plant-growth promotion [65,66]. Genes encoding acid phosphatases, phytases and glucose-1-dehydrogenase (conversion of d-glucose to d-glucono-1,5-lactone) represent the functional context of ‘phosphorus solubilisation’. Likewise, bacteria producing phytohormones or interfering with the plant’s phytohormone metabolism may affect plant growth. For example, 1-aminocyclopropane-1-carboxylate deaminase (AcdS) interferes with the plant’s ethylene synthesis thereby enhancing plant growth [67]. Most of the assembled *acdS* genes were assigned to the *Actinobacteria* (Table S3). Moreover, several genes predicted to function in synthesis of the phytohormone auxin, representing a major plant-growth promoting factor [68,69], were identified. Volatile organic compounds (VOCs) such as acetoin and 2,3-butanediol may induce plant-growth promotion [70,71]. Several enzymes predicted to be involved in VOC synthesis are encoded by different species of the LTE-1 microbiome. Genes for trans-2,3-dihydro-3-hydroxyanthranilate isomerases of the phenazine biosynthesis pathway were detected among the assembled genes. Phenazines feature antimicrobial activity and therefore potentially may counteract phytopathogens [72], thus contributing to plant health. Differential abundance analyses concerning PGP marker genes revealed no differences regarding soil treatments (see Figure S10). It may be speculated that differential abundance of particular PGP-species in response to soil treatments is masked by non-responding microbiome members of the same functionality when functional profiles are considered (see below).

### 3.4. Binning of Metagenomically Assembled Contigs to Access the Most Prominent Genomes of the LTE-1 Soil Microbiome

In order to acquire genome sequence information of abundant LTE-1 microorganisms, contigs assembled from metagenome reads were binned to MAGs (Metagenomically Assembled Genomes) providing the basis for further characterization of abundant soil microbiome members and reconstruction of their metabolism. Each individual metagenome dataset representing a particular soil cultivation and treatment scheme was mapped on assembled contigs to determine their sequence coverage. Contig binning relies on coverage information and tetra-nucleotide frequencies of contigs [32,33]. Merging of the MetaBAT and MaxBin bins by application of DAS Tool yielded 14 MAGs, out of which ten represent high-quality MAGs (see Table 2). These feature estimated completenesses of 57% to 97% and mostly low contamination rates as determined by means of CheckM (see Table 2). Of these ten MAGs, four MAGs represent the kingdom *Archaea* and six belong to the kingdom *Bacteria*. Classification on lower taxonomic ranks by means of the Lowest Common Ancestor (LCA) approach of predicted genes within MAGs showed that all four archaeal MAGs belong to the phylum *Thaumarchaeota*. Taxonomic classifications for all MAGs are provided in Table 2.

### 3.5. Functional Characterization of Bacterial MAGs

To deduce the functional potential of MAGs, metabolic reconstruction was performed by mapping of MAG-encoded gene products to KEGG pathways/modules. Moreover, Carbohydrate-active enzymes of MAGs were predicted and secondary metabolite synthesis pathways were explored. Metabolic reconstructions focused on possible plant-beneficial functions of MAGs.

#### 3.5.1. A Predicted Methylotrophic Bacterium of the Order *Rhizobiales* Is Represented by MAG_13

**Figure 4:**
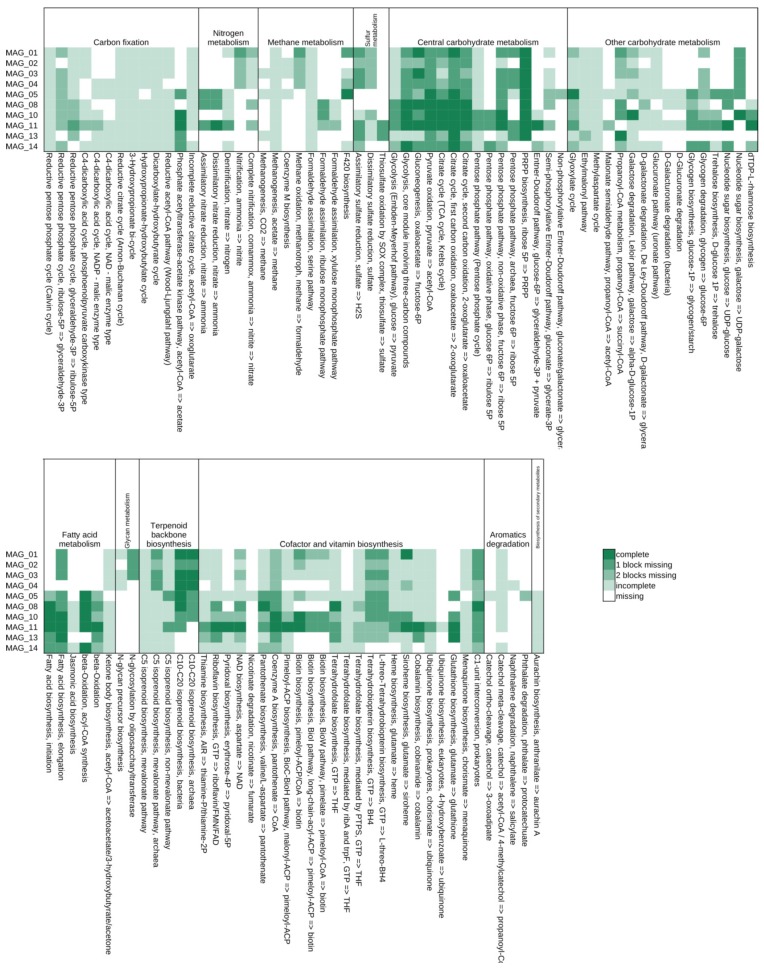
Functional potential of the ten high quality metagenomically assembled genomes (MAGs) as deciphered by means of KEGG (Kyoto Encyclopedia of Genes and Genomes) modules. Rows represent MAGs; KEGG modules are arranged in columns and grouped by superordinate category. Colors of the heatmap indicate the completeness level of identified KEGG modules.

**Figure 5:**
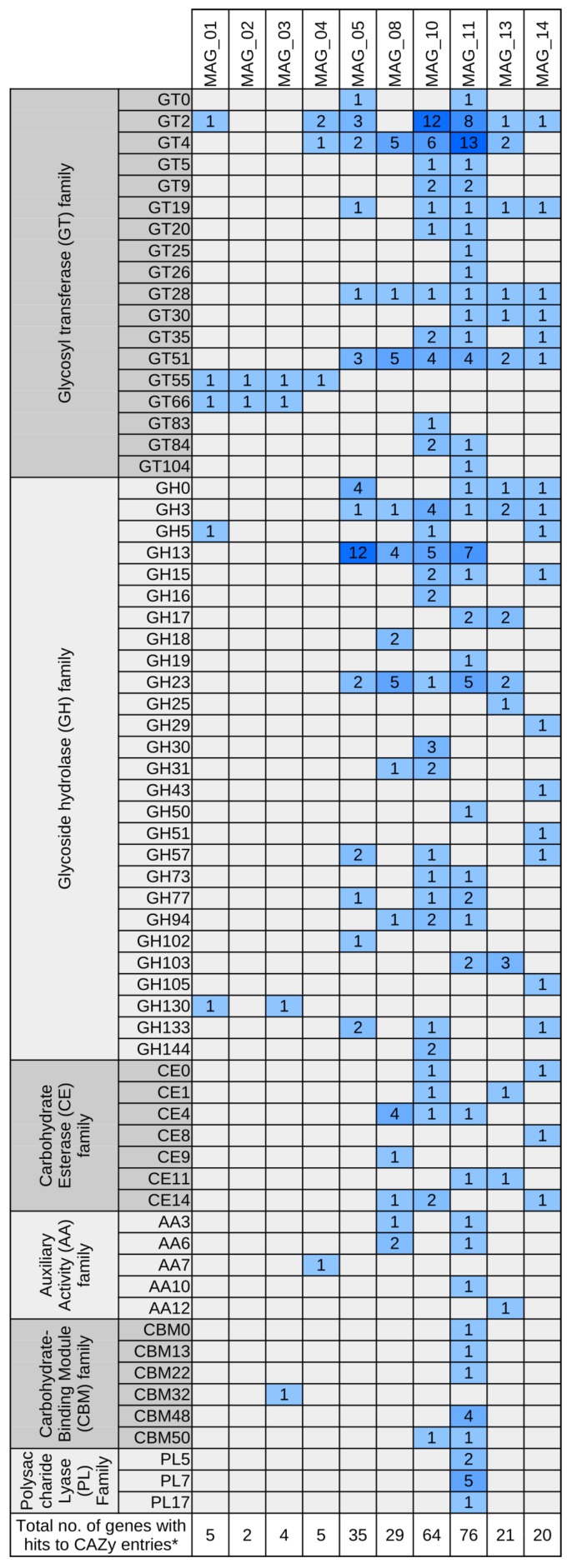
Carbohydrate-active enzyme (CAZy) families identified in the ten high quality metagenomically assembled genomes (MAGs) as deciphered by means of dbCAN2 *in silico* screening (dbCAN2—web server for automated Carbohydrate-active enzyme ANnotation). Columns represent the MAGs; rows are blocked by CAZy modules and further partitioned into families of structurally-related catalytic and carbohydrate-binding modules (or functional domains) of enzymes that degrade, modify, or create glycosidic bonds (http://www.cazy.org/). Colors of the heatmap indicate the number of genes assigned to the respective CAZy family.

MAG_13 was assigned to the order *Rhizobiales* (class *Alphaproteobacteria*). Approximately, 43% of its genes have their closest homologues in the genus *Methyloceanibacter* comprising marine methylotrophic species from i.a., North Sea sediments [73]. Methylotrophs are key players in global carbon cycling [74,75]. A gene encoding methanol dehydrogenase is not present in MAG_13. However, the metagenomically assembled LTE-1 gene set comprises methanol dehydrogenase (EC 1.1.2.7) genes originating from *Rhizobiales* and in particular from the genus *Methyloceanibacter*, indicating that methylotrophic *alpha-Proteobacteria* are present in the soil habitat analyzed. Interestingly, MAG_13 encodes pyrroloquinoline-quinone synthase (EC 1.3.3.11) involved in synthesis of the prosthetic group of methanol dehydrogenase. Moreover, formylmethanofuran-tetrahydromethanopterin N-formyltransferase (EC 2.3.1.101) genes assigned to *Methyloceanibacter* are present in the assembled gene set. The latter enzyme also plays a key role in C1-metabolism. Further enzymes of C1-metabolism such as methenyltetrahydromethanopterin cyclohydrolase (EC 3.5.4.27), 5,6,7,8-tetrahydromethanopterin hydro-lyase (EC 4.2.1.147), glycine hydroxymethyltransferase (EC 2.1.2.1) and formate dehydrogenase (EC 1.17.1.9) are encoded in MAG_13. Genome analyses revealed that MAG_13 presumably assimilates ammonium via glutamine synthetase (EC 6.3.1.2) and glutamate synthase (EC 1.4.1.14) but also encodes a cyanase (EC 4.2.1.104) converting cyanate to carbamate and a nitronate monooxygenase (EC 1.13.12.16) for detoxification of nitro-toxins such as propionate-3-nitronate or utilization of corresponding compounds as a nitrogen source. Some methylotrophic bacteria produce 1-aminocyclopropane-1-carboxylate (ACC) deaminase interfering with the plant’s ethylene metabolism [76]. Several ACC deaminase genes (*acdS*, see Table S3) identified in the LTE-1 metagenome were assigned to unclassified *Rhizobiales* suggesting that corresponding species may be active in plant-growth promotion. Further properties specified by MAG_13 are listed in Table 2 and Figure 4 and Figure 5.

To summarize, the soil *Rhizobiales* species represented by MAG_13 seems to share genomic traits with marine bacteria of the genus *Methyloceanibacter* including methylotrophy and metabolism of C1 components. Management of these and related soil species presumably is of importance regarding soil carbon cycling and plant productivity, since it was reported that root growth promotion is a characteristic feature of the order *Rhizobiales* as shown at the example of the *Arabidopsis thaliana* model system [77]. Moreover, the relevance of methylotrophic bacteria in sustainable agriculture has recently been reviewed [76].

#### 3.5.2. A Putative Plant-Growth-Promoting *Pseudomonas* Species Is Represented by MAG_11

MAG_11 assigned to the genus *Pseudomonas* (class *Gammaproteobacteria*) represents the most complete genome bin (97.4%) and features a very low contamination rate of 4.7%, as determined based on presence or absence of single copy marker genes. The genus *Pseudomonas* is amongst the most abundant genera identified in the LTE-1 metagenome (Figure 2b). Particular soil- and plant-associated pseudomonads are known for their biocontrol activities and plant-growth-promoting (PGP) properties [78]. Hence, they are of interest for application in alternative plant cultivation concepts and for protection of crops against pathogens. Most of the genes harbored by MAG_11 were classified as belonging to the preliminary taxon ‘unclassified *Pseudomonas*’. Enzymes of the KEGG Tryptophan Metabolism (00380) potentially involved in indole-acetic acid (IAA, auxin) synthesis were identified. In particular, genes for an aldehyde dehydrogenase (NAD+) (EC 1.2.1.3), amidase (EC 3.5.1.4) and monoamine oxidase (EC 1.4.3.4) are present. Production of the phytohormone IAA has to be considered as major plant-growth-promoting feature of plant-beneficial bacteria [63]. Likewise, the *Pseudomonas* species represented by MAG_11 has the capacity to synthesize gibberellins (GAs) since enzymes catalyzing production of geranyl–pyrophosphate and geranyl–geranyl–pyrophosphate are encoded in its genome. The latter metabolite is a precursor of diterpenoid biosynthesis also including GAs that may function in plant growth regulation. Bacterial production of both, IAA and GAs is supposed to efficiently stimulate plant growth [79].

**Figure 6:**
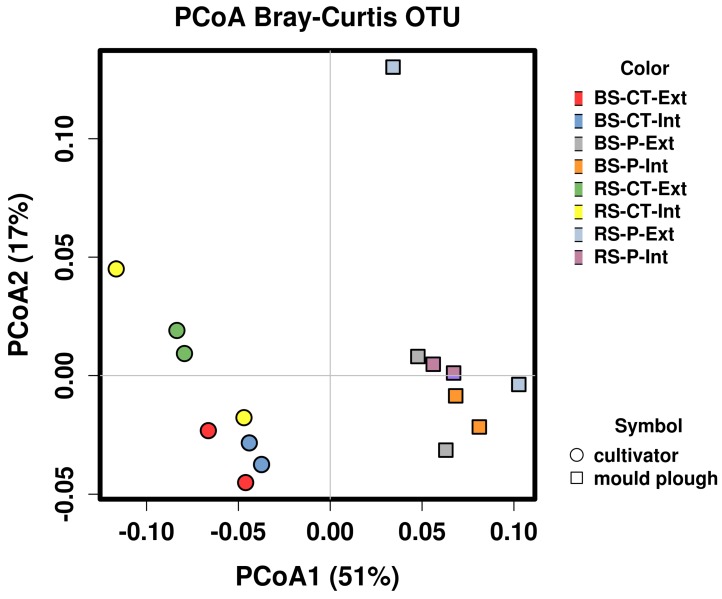
Principal coordinate analysis of abundance data obtained for 162 metagenomically assembled genomes (MAGs) of the soil microbial communities analyzed. The scatter plot shows principal coordinate axis 1 (PCoA1) versus principal coordinate axis 2 (PCoA2) that explain 51% and 17% of the total variance, respectively. In addition, 162 MAGs (>20% completeness and ≤15% contamination by means of checkM) were used for this analysis. Cultivator-treated samples are represented by circles, mould-plough treated samples by rectangles. Abbreviations stand for bulk soil (BS) or root-affected soil (RS), tillage by mould-board plough (P) or cultivator (CT), and nitrogen fertilization intensive (Int) or extensive (Ext).

Interestingly, MAG_11 possesses all genes necessary for production of poly(3-hydroxybutyrate) (PHB) from acetyl-CoA via acetoacetyl-CoA and (R)-3-hydroxybutyryl-CoA. PHB represents a carbon and energy resource accumulated within the cell under unfavorable environmental conditions enhancing competitiveness and survivability of the bacterium when nutrients are limited [80]. Transcriptional upregulation of the PHB biosynthesis pathway has been shown for the microbiome of the wheat root vicinity [81] substantiating importance of this pathway for plant-associated bacteria. MAG_11 completely covers the KEGG module ‘Dissimilatory nitrate reduction’ (Figure 6) and therefore is most probably able to provide soluble ammonium to host plants by anaerobic respiration. Screening for gene clusters coding for the synthesis of secondary metabolites by means of antiSMASH [40] yielded an extensive array of corresponding clusters in MAG_11. Secondary metabolites are structurally highly diverse compounds that exert various biological effects and have multiple functions in microbial soil communities [82]. Two of ten identified antiSMASH clusters were predicted to encode the biosynthesis of siderophores since they are similar to the Xanthoferrin biosynthesis gene cluster of *Xanthomonas oryzae*. Siderophores scavenge iron from the environment, making it bioavailable to the cell. Furthermore, non-canonical roles of siderophores have been suggested, including modulation of host functions [83]. Moreover, three Arylpolyene-type clusters and an Arylpolyene-Resorcinol-type cluster were predicted. The products of gene clusters from the large and unexplored aryl polyene biosynthetic gene cluster family are believed to play an important role in Gram-negative cell biology [84]. Aryl polyene/dialkylresorcinol hybrid pigments of the *beta-Proteobacterium Variovorax paradoxus* have been found to be functionally related to antioxidative carotenoids and most probably have a protective function [85]. In addition, two bacteriocin clusters were detected. Bacteriocins are small antimicrobial toxins contributing to the competitiveness of the producing bacterium [86]. Most interestingly, a hybrid Homoserine lactone butyrolactone cluster and a ladderane cluster with similarity to the burkholderic acid (malleilactone) cluster were predicted. Lactones generally are microbial volatiles; volatile butyrolactones exhibit antibacterial activity [87]. Homoserine lactones are a widely distributed class of quorum sensing molecules, for some of which antibacterial properties were reported [88]. Burkholderic acid is a polyketide synthase-derived cytotoxic siderophore that exhibits antibacterial activity [89,90]. Figure 5 shows the predicted Carbohydrate Active enZymes (CAZy) of the bacterial MAGs. The CAZyme profile of MAG_11 reveals an extensive array of Glycosyl Transferase (GT) family enzymes and Carbohydrate-Binding Module family enzymes. Furthermore, it possesses predicted enzymes categorized into the Polysaccharide Lyase families PL5, PL7 and PL17, which are associated with alginate lyases and therefore might indicate the ability of MAG_11 to form biofilms.

#### 3.5.3. A Member of the Candidate Order ‘Unclassified *Deltaproteobacteria*’ Predicted to Encode the DOXP/MEP Isoprenoid Biosynthesis Pathway Is Represented by MAG_05

MAG_05 represents a member of the candidate order ‘unclassified *Deltaproteobacteria*’. Basic genome features of this MAG are depicted in Table 2. Interestingly, MAG_05 covers many KEGG modules of the terpenoid backbone synthesis pathway (Figure 4). It possesses genes for almost all enzymes of the non-mevalonate isoprenoid biosynthesis pathway DOXP/MEP (1-deoxy-D-xylulose 5-phosphate/2-C-methyl-D-erythritol 4-phosphate) starting with the condensation of pyruvate and glyceraldehyde-3-phosphate and finally yielding precursors of isoprenoid synthesis such as isopentenyl-diphosphate, geranyl-diphosphate, farnesyl-diphosphate and geranyl–geranyl-diphosphate [91]. In particular, the latter metabolite is needed for diterpenoid biosynthesis also comprising gibberellin phytohormones. Bacterial hormone production may interfere with plant growth regulation. Likewise, enzymes involved in conversion of indole compounds including the phytohormone IAA are encoded in MAG_05. Moreover, genes for enzymes (EC 2.2.1.6 and EC 1.1.1.76) potentially involved in synthesis of the volatile organic compounds (VOCs) acetoin and 2,3-butanediol were identified. VOC production may stimulate plant growth and induce systemic resistance [63]. Furthermore, two clusters for biosynthesis of secondary metabolites were identified. The predicted product of the first cluster is a terpene. Its core biosynthetic genes presumably encode a phytoene synthetase (EC 2.5.1.32) and malto-oligosyltrehalose trehalohydrolase (EC 3.2.1.141). The second cluster belongs to the type arylpolyene and its core biosynthetic genes were annotated to encode a beta-ketoacyl synthetase (EC 2.3.1.41) and 3-deoxy-D-manno-octulosonic acid transferase (EC 2.4.99.12). Concerning its nitrogen metabolism, MAG_05 encodes the enzymes glutamate dehydrogenase, glutamine synthetase and glutamate synthase for synthesis and interconversion of the amino acids glutamate and glutamine. Moreover, genes for dissimilatory nitrate reduction and the denitrification enzyme nitric oxide reductase (EC 1.7.2.5) catalyzing reduction of nitric oxide (NO) to nitrous oxide (N_2_O) were identified. Nitrogen oxides are known to act as signaling molecules in plants [92]. Predictably, carbohydrates are metabolized via glycolysis and the citrate cycle utilizing oxygen as terminal electron acceptor (aerobic metabolism) since all necessary enzymes of these pathways are encoded in MAG_05. By means of dbCAN2, a very high number of genetic determinants of starch degradation was predicted (GH13). Saprophytic, but also rhizosphere bacteria comprise starch degrading species, since plant tissues and especially the rhizosphere are rich in starch substrate [93].

#### 3.5.4. A Putative Plant-Growth-Promoting Bacterium of the Family *Bacillaceae* Is Represented by MAG_08

MAG_08 was classified to the family *Bacillaceae* (phylum *Firmicutes*) with 70.4% of its genes being assigned to the tentative taxon ‘unclassified *Bacillaceae*’ at the genus level. Members of the family *Bacillaceae* are mostly aerobic heterotrophic saprophytes well known for their ecosystem functions in their primary habitat soil [94,95]. These functions include plant–growth–promotion and biocontrol, as well as functions in the carbon and nitrogen cycle. Therefore, MAG_08 was searched for genes featuring functions in this context. Figure 4 shows that MAG_08 at least partially covers the KEGG pathway ‘complete nitrification, comammox’ and therefore might be able to oxidize both ammonia and nitrite. MAG_08 might be able to stimulate plant growth since key genes coding for enzymes needed for the biosynthesis of phytohormones and VOCs are present. Reconstruction of KEGG pathways revealed relatively high numbers of hits in the maps ‘Biosynthesis of secondary metabolites’ (150) and ‘Biosynthesis of antibiotics’ (110). Three secondary metabolite gene clusters were predicted by application of antiSMASH [40]. An NRPS-type cluster, a Terpene-type cluster and a ‘type III polyketide synthase (PKS)’ cluster were identified. Furthermore, a key determinant of the *Bacillaceae* lifestyle is ‘social’ behavior, which includes cell-to-cell signaling via quorum sensing. In this context, 26 predicted features were assigned to the KEGG map 02024 ‘Quorum sensing’. The carbohydrate-active enzyme (CAZy) profile of MAG_08 (see Figure 5) as determined by application of dbCAN2 [38] disclosed the Glycoside Hydrolases (GH) families GH3, GH13, GH18 (chitin degradation), GH23 (peptidoglycan hydrolase family), GH31, associated with hemicellulase activity and GH94 (cellulose degradation). Therefore, the species represented by MAG_08 may play a role in decomposition of organic matter in soil.

#### 3.5.5. A Member of the *Flavobacteriaceae* Predicted to Decompose Complex Carbohydrates Is Represented by MAG_10

MAG_10 was classified as member of the family *Flavobacteriaceae* (phylum *Bacteroidetes*). It was predicted to be nearly complete and features a low contamination rate (Table 2). Members of the *Bacteroidetes* have previously been linked to decomposition of plant biomass in different terrestrial environments [96]. The dbCAN2 profile of MAG_10 stands out in richness and composition compared to the other bacterial MAGs (see Figure 5). It includes potentially hemicellulolytic enzymes of the Glycoside Hydrolase families GH16, GH30, GH5, GH30 and Carbohydrate-Binding Module family CBM50, which have been linked to degradation of cellulose. Presence of enzymes belonging to these CAZyme families, identified in cellulolytic soil bacteria, suggests that MAG_10 represents a typical cellulose decomposer [96,97]. Moreover, the rather new family GH144 was predicted in MAG_10. Enzymes of GH144 prefer β-1,2-glucan as substrate. β-1,2-Glucan is a polysaccharide produced by Gram-negative bacteria with important roles in infection and symbiosis [98,99]. The metabolic profile of MAG_10 (Figure 6) also suggests its capability of metabolizing many different carbohydrates.

Members of the *Flavobacteriaceae* have previously been linked to plant disease suppressiveness [100]. Three secondary metabolite clusters were identified in MAG_10 applying antiSMASH. An arylpolyene-type cluster was predicted. Many bacteria interacting with higher plants harbor arylpolyene-type biosynthetic gene clusters (BGCs) [84]. Furthermore, MAG_10 was predicted to encode a terpene producing biosynthetic gene cluster. The production of several terpenes has been observed in plant-growth promoting bacteria (PGPB), some of which also induce the plant cell production of terpenes, enhancing plant growth and yield [101]. The third antiSMASH cluster was classified as type III polyketide synthase (PKS). Microbial type III PKSs are involved in the biosynthesis of various compounds such as chalcones, pyrones, acridones, phloroglucinols, stilbenes, and resorcinolic lipids featuring different biological functions [102,103]. Presence of secondary metabolite synthesis clusters suggests a possible role in plant–growth–promotion for MAG_10.

#### 3.5.6. A Member of the Phylum *Acidobacteria* Predicted to Be Versatile in Transport Activities and Carbohydrate Utilization Is Represented by MAG_14

MAG_14 of the phylum *Acidobacteria* is of particular interest since species of this phylum represent important members of soil microbiomes [104,105]. Abundance of this phylum was already apparent from evaluation of taxonomic profiles based on single read analyses (see above). Only few acidobacterial genomes were completely sequenced so far which is due to the fact that many *Acidobacteria* pose challenges regarding their cultivation. Genome analyses revealed that most *Acidobacteria* are versatile in transport activities and carbohydrate and protein utilization [104,105]. General genome features of MAG_14 are depicted in Table 2 and Figure 4. Although this MAG represents a comparatively incomplete genome bin (completeness of 56.8%), it encodes several enzymes predicted to be involved in carbohydrate utilization. Different genes for enzymes of the Glycosyl-Hydrolase (GH) group EC 3.2.1.- were identified. Likewise, the Carbohydrate-Active enZYmes (CAZy) profile of MAG_14 comprises several GH-family entries as depicted in Figure 5 (http://www.cazy.org/Glycoside-Hydrolases.html). MAG_14 also encodes different serine endopeptidases (EC 3.4.21.-) and, accordingly, the corresponding bacterium may be active in proteolysis. Presence of genes specifying putative oligopeptide ABC-transporter components supports the latter prediction. Another ABC-transporter represents an Fhu-homologous siderophore-iron transport system that most likely is of importance for the host since iron frequently is a limiting resource in the soil environment. The genome bin encodes nitrogen metabolism genes for nitric-oxide reductase (EC 1.7.2.5, a denitrification enzyme) and glutamate synthase (EC 1.4.1.13/14) functioning in conversion of glutamine to glutamate. Lack of further nitrogen metabolism genes may be due to incompleteness of MAG_14. *Acidobacteria* commonly feature an aerobic energy metabolism. MAG_14 encodes a cytochrome c oxidase but also components of the cytochrome bd complex (CydA/B) most likely possessing a higher affinity for oxygen and thus allowing transfer of electrons to oxygen under microaerobic conditions. Accordingly, the bacterium represented by MAG_14 may be competitive in niches with reduced oxygen availability. In agreement with this, it was previously reported that many acidobacterial species seem to be able to respire oxygen under microaerophilic conditions [104,105].

Similar to MAG_05, MAG_14 possesses genes for almost all enzymes (six of eight) of the isoprenoid biosynthesis pathway DOXP/MEP (1-deoxy-D-xylulose 5-phosphate/2-C-methyl-D-erythritol 4-phosphate) starting with the condensation of pyruvate and glyceraldehyde-3-phosphate and finally yielding precursors of isoprenoid synthesis such as geranyl-diphosphate, farnesyl-diphosphate and geranyl–geranyl-diphosphate [91]. The latter metabolite may feed diterpenoid biosynthesis also comprising gibberellic acid involved in regulation of plant growth and development. Likewise, MAG_14 may be able to synthesize the auxin IAA (indole-3-acetic acid) since genes for relevant enzymes (EC 1.4.3.4 and EC 1.2.1.3) of the tryptophan metabolism (KEGG map00380) were identified. Previously, *Acidobacteria* subdivision 1 strains were reported to promote plant growth presumably involving synthesis of the plant hormone IAA [106].

### 3.6. Functional Characterization of Archaeal MAGs

All archaeal MAGs (MAG_01, MAG_02, MAG_03 and MAG_04) were assigned to the phylum *Thaumarchaeota* representing a relatively young and scarcely investigated taxon comprising archaeal ammonia oxidizers and many species with unknown energy metabolism [107]. According to the GTDB taxonomy [36], the archaeal MAGs were assigned at lower taxonomic ranks (see Table 2). MAG_01, MAG_02 and MAG_03 belong to the family *Nitrososphaeraceae* and MAG_04 to the genus *Nitrososphaera* as determined by means of GTDBtk (https://github.com/Ecogenomics/GtdbTk).

#### A Putative Ammonia Oxidizer of the Phylum *Thaumarchaeota* Is Represented by MAG_01

MAG_01 encodes the key nitrification enzyme ammonia monooxygenase (EC 1.14.99.39) converting ammonia to hydroxylamine. The reference species of the genus *Nitrososphaera* was only recently discovered in soil [108] and members of this genus are known to oxidize ammonia. They were reported to have an important function in nitrogen cycling in marine and terrestrial ecosystems [109,110]. Moreover, *Nitrososphaera* species feature an autotrophic metabolism since they are able to fix carbon dioxide (CO_2_) for biosynthesis of organic molecules [111]. Carbon fixation is of importance regarding the atmospheric carbon dioxide balance. To fix CO_2_, they utilize a specific pathway, the so-called 3-hydroxypropionate/4-hydroxybutyrate autotrophic carbon dioxide assimilation pathway of *Archaea* [112]. Most of the enzymes involved in this pathway (15 of a total of 18) are encoded in MAG_01. In particular, genes for the enzymes acetyl-CoA carboxylase and propionyl-CoA carboxylase incorporating HCO_3_^−^ into organic molecules and the key enzyme 4-hydroxybutanoyl-CoA dehydratase (EC 4.2.1.120) were identified. Accordingly, the species represented by MAG_01 combines ammonia oxidation and carbon dioxide fixation and hence seems to play an important role in the nitrogen cycle and carbon sequestration within the agricultural soil environment analyzed. Moreover, MAG_01 possesses genes for enzymes of the propanoate metabolism involved in conversion of propanoate to succinate or vice versa. Commonly, the volatile fatty acid (VFA) propanoate is formed during anaerobic digestion of biomass mainly by members of the *Firmicutes* [113] that were also detected in taxonomic profiles of the LTE-1 field plots (see above).

Interestingly, members of the *Thaumarchaeota* are discussed to interact with plants since some of them feature an endophytic lifestyle [114]. MAG_01 encodes enzymes for geranyl–pyrophosphate and geranyl–geranyl–pyrophosphate synthesis depicted in the terpenoid backbone biosynthesis pathway (KEGG map 00900). The latter metabolite is a precursor for synthesis of gibberellins (diterpenoid biosynthesis) that function as phytohormones [115]. This finding may suggest a role of the corresponding archaeon in microbe–plant interaction. A previous study reported on a positive correlation of *Nitrososphaera* species to field sites under agricultural cultivation [45]. Since the class *Nitrososphaeria* is amongst the top ten of the most abundant classes of the soil microbiome analyzed (relative abundances of 1.23 to 1.95%), soil management practices affecting abundances of *Nitrososphaeria* members are of importance (see below). The other MAGs assigned to the family *Nitrososphaeraceae* (class *Nitrososphaeria*) feature similar characteristics as compared to MAG_01 (see Figure 4 and Figure 5).

### 3.7. Differentially Abundant MAGs of the Soil Microbiome in Relation to Tillage and Fertilization Regimes

To determine the relative abundance of MAGs as a response to the applied soil management practices, all 16 metagenome datasets were mapped back to each contig of the assembled soil metagenome dataset. The raw read counts of contigs forming a MAG were summed up and normalized. Resulting RPKM (Reads Per Kilobase per Million) values represent MAG abundances under different soil conditions. In total, 162 MAGs (>20% completeness and ≤15% contamination as determined by means of CheckM) were considered for abundance analyses. Clustering of MAG abundance data (Figure S12) divided the samples into two groups and three single samples (RS-P-Ext_R1 and _R2 and RS-CT-Int_R1). Clusters representing mould-board plough tillage (P) and cultivator tillage (CT) samples emerge as distinguishable groups (see Figure S12). This separation regarding tillage practice was confirmed by ANalysis Of SIMilarity (ANOSIM) testing, also showing that the MAG abundance profiles are most distinct in response to the factor tillage (Bray–Curtis analysis of similarity, ANOSIM R = 0.769, *P* = 0.001). In contrast, the soil compartment (BS vs. RS) and fertilization/pesticide application intensity (Int vs. Ext) only marginally shaped MAG abundance profiles (Bray–Curtis analysis of similarity, ANOSIM R = 0.1 and 0.028, *P* = 0.13 and *P* = 0.263, respectively). Moreover, a clear demarcation regarding abundance of MAGs between mould plough- and cultivator-treated soil samples was apparent along principal coordinate axis 1 (PCoA1) of the PCoA plot shown in Figure 6. Along principal coordinate axis 2 (PCoA2), a separation in terms of the compartment (BS vs. RS) was also apparent. Clustering results based on MAG abundances were in accordance considering principle patterns in corresponding analyses based on single-read taxonomic classifications (shown in Supplemental Figure S9). As expected, the most complete MAGs (as reported in Table 2) show the highest abundances, except for MAG_14 (*Acidobacteria*). This is in accordance with the other 28 MAGs classified as *Acidobacteria*, which all feature a moderate abundance.

**Figure 7:**
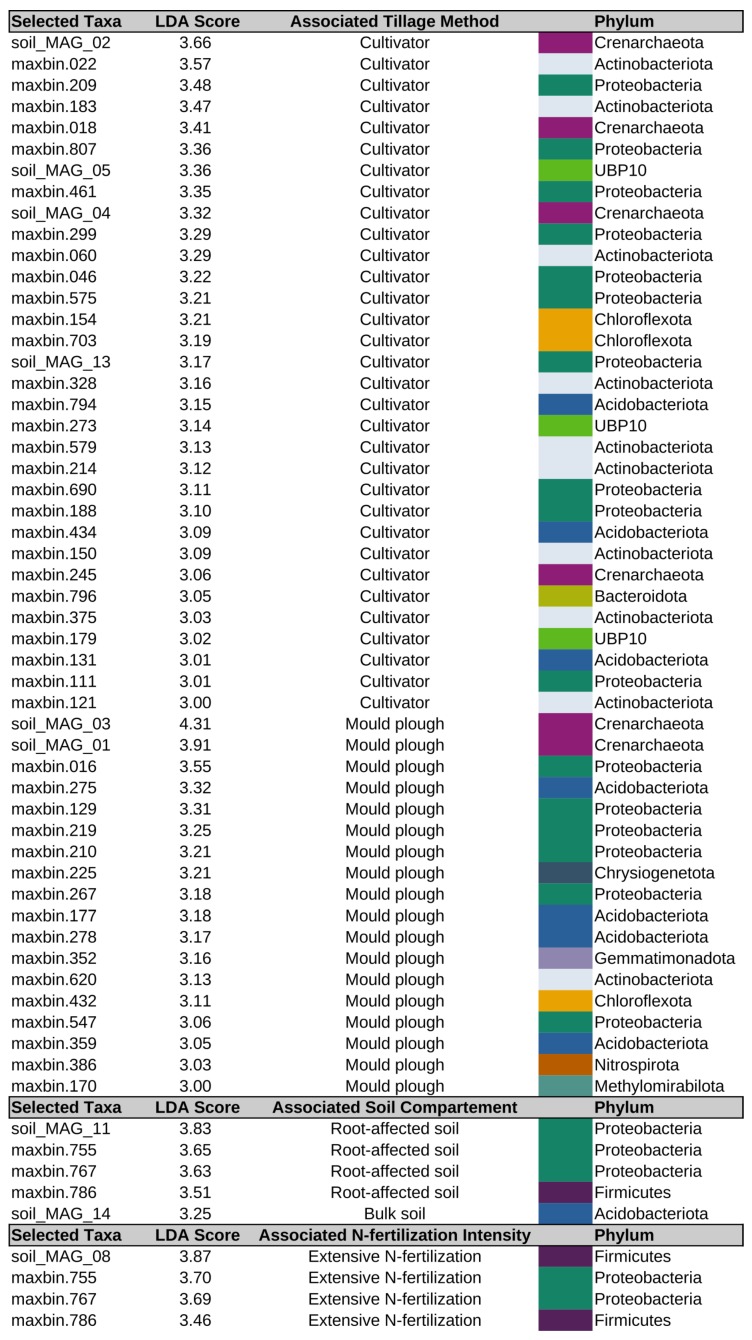
Significantly differentially abundant MAGs. For linear discriminant analysis (LDA) effect size method (LEfSe) [43] analysis, data was grouped by tillage, compartment (bulk soil and root-affected soil) and fertilization. LDA scores estimate the effect size and therefore indicate the degree of consistent difference in relative abundance between features in the two classes of analyzed microbial communities [43]. Color indicates the phylum of each MAG as assigned by GTDBtk (https://github.com/Ecogenomics/GtdbTk; *Crenarchaeota* corresponds to *Thaumarchaeota* in the NCBI taxonomy).

Strikingly, many *Thaumarchaeota* MAGs (*Crenarchaeota* according to the GTDB taxonomy) cluster together by abundance profiles (Figure S12). Moreover, the best assembled *Thaumarchaeota* MAGs (MAG_01, MAG_02, MAG_03 and MAG_04) have very high abundances in all samples, with MAG_01 occurring with the highest reported abundances of all MAGs. Most strikingly, MAG_01 and MAG_03 (both family *Nitrososphaeraceae*) respond to the tillage practice, as they show remarkably higher abundances in mould-board ploughed samples (P) compared to those treated with cultivator (CT) (Figure S12). In order to confirm these observations, MAGs most likely explaining differences between soil management practices were identified using the linear discriminant analysis (LDA) effect size method (LEfSe) [43]. Standard tests for statistical significance are implemented within LEfSe and are coupled to additional tests addressing biological consistency and effect relevance. The results of the LEfSe analyses blocked by tillage method, soil compartment and N-fertilization intensity are shown in Figure 7. Strikingly, archaeal MAGs exclusively respond to tillage practice. LEfSe analysis results further confirm the differential abundances of MAG_03 and MAG_01 in the associated group ‘mould plough’ with the highest overall effect sizes (4.3 and 3.9 orders of magnitude, respectively; see Figure 7), reflecting remarkable abundance in samples treated with mould plough and consistently lower abundances in cultivator treated samples. MAG_03 and MAG_01 both were predicted to possess a glycoside hydrolase of family GH130, which contains phosphorylases and a glycoside hydrolase acting on β-mannosides, which are major components of plant structural polysaccharides [116]. In mould plough tillage, remaining crop residues are ploughed in by soil inversion. The ability to degrade plant cell walls could explain the differential abundance of these two *Thaumarchaeota* members in soils under mould plough tillage. Likewise, MAG_02 and MAG_04, which respond to tillage by cultivator, represent members of the *Thaumarchaeota*. A recent study investigated environmental variables affecting the relative abundance of *Thaumarchaeota* in soil [117]. The authors showed that the total nitrogen content appeared to be the environmental variable that affected relative abundance estimates of *Thaumarchaeota* most strongly. In the present study, it appeared that the phylum *Thaumarchaeota* did not respond differentially to the nitrogen fertilization regime, but to the tillage practice, corroborating previous results which indicate greater abundances of *Thaumarchaeota* members (*Nitrosophaera*) in response to agriculture in general [45]. Interestingly, the genus *Nitrososphaera* is among the most abundant genera of the LTE-1 microbiome according to taxonomic profiling based on single reads (see Figure 2b).

MAG_02, MAG_ 05, MAG_04 and MAG_13 are associated with tillage by cultivator (CT) (see Figure 7). Due to their genetic potential regarding soil-health-ameliorating and plant-growth promoting determinants, MAG_05 and MAG_13 can be considered as indicators for a healthy soil. Thus, it can be deduced that cultivator tillage is advantageous, since its application favors beneficial species.

Regarding the effect of the soil compartment, according to the LEfSe analysis, MAG_11 (genus *Pseudomonas*) explains differences related to root-affected soil (RS). Since the genus *Pseudomonas* comprises many well-known PGPB, and the functional characterization of MAG_11 provides evidence for its plant-growth promoting potential, this result meets expectations. Interestingly, MAG_11 occurred with a peaking high abundance in sample RS-CT-Int_R1, most probably reflecting soil sample heterogeneity. Differential abundance of MAG_14 (*Acidobacteria*) correlated with bulk soil (BS). This is in accordance with the findings that *Acidobacteria* are among the most dominant phyla in soil-borne microbial communities [118].

Grouping by N-fertilization intensity, LEfSe analysis revealed that MAG_08 (*Bacillaceae*) responded to reduced N-fertilization with a high effect size (LDA score of 3.87, see Figure 7). Members of the *Bacillaceae* are commonly known to play key roles in nitrogen-cycling processes and include nitrogen fixers and denitrifiers [94]. The latter most probably applies to MAG_08 based on its genetic potential. Strikingly, maxbin.755 and maxbin.767 (both assigned to the genus *Acidovorax* according to the GTDB taxonomy), as well as maxbin.786 (order *Mycoplasmatales*), responded to both root-affected soil and extensive N-fertilization. Members of the genus *Acidovorax* have previously been shown to be associated with the rhizosphere [119], which would explain its enrichment in root-affected soil. Furthermore, *Acidovorax* members are known as denitrifiers, which are able to directly degrade lignocellulosic biomass as carbon sources [120]. *Mycoplasmatales* members are cell wall-less bacteria found to be generally associated with arthropod or plant hosts, or with ruminants [121], but also endosymbiotic members hosted by plant-symbiotic, arbuscular mycorrhizal fungi (AMF) are known [122]. Such a lifestyle might explain the association of maxbin.786 to both root-affected soil and reduced N-fertilization, since it presumably persists in spatial proximity to plants. Furthermore, since the abundance of AMF was shown to strongly decline in ecosystems where N is available in excess, the endophyte’s abundance is most likely to decline accordingly [123].

In summary, the tillage practice has the most pronounced effect on abundances of particular soil community members, whereas the soil compartment (‘bulk soil’ vs. ‘root-affected soil’) and fertilization intensities appeared to be of minor importance in the present study. Nevertheless, for all factors taken into consideration, an effect on abundant particular community members represented by MAGs was reported.

## 4. Conclusions

This study provides deeper insights into the taxonomic composition and functional profile of the microbiome residing in the loess-chernozem soil of the LTE-1 field site at Bernburg (‘Magdeburger Börde’, Saxony-Anhalt, Germany) as deduced from thorough metagenome analyses. Effects of long-term farming practices, namely ploughing techniques combined with fertilization intensities on the microbiome composition and functionalities, were within the focus of the experimental setup. To the best of our knowledge, so far, microbiome analyses based on metagenome sequencing have not been conducted for a loess-chernozem soil type representing one of the most fertile soils in Germany. Therefore, obtained metagenome sequence data and assembled gene sets represent a valuable reference repository, in particular for the analyzed soil type but also for agricultural soils in general. Taxonomic profiling based on shotgun metagenome sequencing of whole community DNA instead of 16S rRNA gene amplicon sequencing provides a comparatively unbiased insight into the composition of the soil microbiome because the latter method involves primer- and PCR-biases. Taxonomic community profiles show a high degree of compositional similarity with regard to farming practices. Likewise, important potential functions and predicted metabolic pathways of microbiome members were deduced and are represented by nearly 7 million recovered genes. Among these, numerous potential plant-beneficial, plant-growth-promoting and biocontrol traits predicted to have functions in nutrient provision, phytohormone synthesis or modulation of hormone levels, antagonism towards phytopathogens and signaling in microbe–plant interactions were obtained. Taxonomic classifications suggest a wide distribution of corresponding genes among soil microbiome members. So far, non-recognized species may contribute to this functional context. However, evidence that plant-beneficial traits were enriched depending on the applied soil management strategies was not obtained. It is assumed that farming practice effects on specific microbiome members are masked by other non-responding taxa featuring similar functionalities. Due to the high diversity of agricultural soil microbiomes, high redundancy of functions is likely to occur and evidence for this assumption has been obtained by the metagenome analyses of this study. Whole metagenome shotgun sequencing offers a high resolution to identify differentially abundant features between samples. Considering the high diversity in the LTE-1 metagenome, an even higher sequencing depth might be desirable to achieve a resolution facilitating the detection of significant differentially abundant features. Variances between biological replicates may result from heterogeneity of soil samples and probably can be evened by increasing the number of biological replicates. However, metagenome sequencing still is cost-intensive, which has to be considered in experimental setups.

Profound long-term tillage effects on the prokaryotic soil microbiome were not supported by the results obtained in this study except for individual species. In other words, the soil memory effect with respect to tillage practices was not as pronounced as expected in the LTE-1 field-site analyzed. Fertilization intensities appeared to be even less important for the LTE-1 microbiome structure, most probably because extensive fertilization (50%) still means excess of nitrogen compared to natural conditions in soil. It is very likely that soil management practices applied in this study affect metabolic activities of community members which in principle can be verified by metatranscriptome or metaproteome analyses. However, differential metabolic activities of the microbiome in response to changing environmental conditions are transient and reflect persistence of the influencial factor.

Metagenome assembly and binning was conducted to reconstruct genomes of so far unknown and abundant microbiome members. Important functions regarding the nitrogen, carbon and energy metabolism and plant-beneficial traits were identified in ten high-quality MAGs that were analyzed in depth. Obtained genome information for these new candidate plant and/or soil beneficial species may guide the development of rational isolation strategies. If successful, application of isolates as inoculants to improve soil health and plant productivity can be considered. It also appeared that some species represented by MAGs respond to agricultural management practices and accordingly appropriate treatments offer possibilities for microbiome engineering. However, tillage, the most crucial factor for shaping MAG abundances in our experimental approach, may not be the only factor that influences soil/plant health and plant productivity. Effects of organic farming/fertilization, cropping systems, crop rotation (implementation of suitable preceding crops) and amendment additives should be considered in future studies to enhance favorable microbiomes. It has also been suggested to breed crops for their ability to recruit more beneficial microbiomes by means of modifying their rhizodeposition. Specific metabolites in root exudates may attract specific microorganisms. Genome information of plant-beneficial species may help to identify candidate metabolites promoting their proliferation in the rhizosphere. Performance of putative biocontrol strains exemplified by MAGs of this study must be verified in controlled experiments using corresponding isolates. Positive strains featuring biocontrol properties may be considered for the compilation of synthetic consortia regarding applications in plant protection and/or fertilization.

Microbial populations exemplified by MAG_01 (*Thaumarchaeota*) and MAG_13 (*Rhizobiales*) probably are of global importance for soil communities since they were predicted to function in carbon cycling/sequestration and conversion of inorganic nitrogen compounds. They are represented in larger numbers as deduced from taxonomic profiles established in this study. The putative methylotrophic bacterium of the candidate taxon ‘unclassified *Rhizobiales*’ represented by MAG_13 is involved in conversion of C1-components and therefore has an assumed impact on the carbon cycle. Likewise, members of the phylum *Thaumarchaeota* predictably contribute to ammonium oxidation and carbon dioxide fixation. Accordingly, they may positively affect the carbon dioxide balance of the atmosphere, which is of importance regarding global warming caused by carbon dioxide and other greenhouse gas emissions. Hence, soil management strategies should consider effects on functional microbiome groups affecting the carbon sequestration ability of soils to promote a climate resilient agriculture.

## Figures and Tables

**Table 1:** Metadata for the Long-Term-Experiment (LTE-1) field site at Bernburg-Strenzfeld (Saxony-Anhalt, Germany). Data taken from Sommermann et al. [24].

Location	Bernburg-Strenzfeld (LTE-1 field site),
	Saxony-Anhalt, Germany
Latitude/Longitude	51.82° N, 11.70° E
Altitude	80 m (above sea level)
Environment	Agricultural farmland
Cultivated crop	Winter wheat cultivar ‘Pamir’
Soil type	Loess chernozem over limestone
Soil texture	22% clay, 70% silt, 8% sand
Horizon (sampled)	Ap
pH	7.0–7.4
P supply	45–70 mg per kg
K supply	130–185 mg per kg
Average annual temperature (1980–2010)	9.7 °C
Average annual precipitation	511 mm
Crop rotation	Grain maize (*Zea mays*), winter wheat (*Triticum aestivum*),
	winter barley (*Hordeum vulgare*),
	winter rapeseed (*Brassica napus* ssp. Napus), winter wheat
Treatment: N-fertilization	220 kg per ha as ammonium sulfate and calcium ammonium
	nitrate + fungicides and growth regulators as described
	in [24] (intensive—Int) or 90 kg per ha as ammonium sulfate
	and calcium ammonium nitrate (extensive—Ext)
Treatment: Tillage	Ploughed using a mould-board plough (P),
	carrier board with combined alternating ploughshares,
	ploughing depth 30 cm, incl. soil inversion or treated with
	cultivator (CT), 10 cm flat non-inverting soil loosening
History	Long-term field trial started in 1992
Collection date	30 July 2015
Sampling depth	0–25 cm (soil corer)
Physicochemical soil properties	[25]
Basic soil parameters	[24] Table S3
Winter wheat yields of the LTE-1 field plots	Described by [24]

**Table 2:** Summary of high-quality Metagenomically Assembled Genomes (MAGs) compiled from metagenomic sequences of LTE-1 soil samples.

			Comp-	Con-				Abun-	Maximal	Sample
			lete-	tami-	Size			Dance	Abundance	with Highest
MAG ID	LCA ^*a*^	GTDB Taxonomy	ness	nation	[bp]	Contigs	Features ^*b*^	Rank ^*c*^	(RPKM)	Abundance
MAG_01	*Thaumarchaeota*	*Nitrososphaeraceae*	88.69	4.85	1,828,699	692	2570	1	7.614	BS-P-Int_R2
MAG_02	*Thaumarchaeota*	*Nitrososphaeraceae*	61.58	7.28	1,705,306	1021	2486	2	5.0159	BS-CT-Int_R1
MAG_03	*Thaumarchaeota*	*Nitrososphaeraceae*	81.37	0.97	1,526,325	556	2279	7	3.5153	BS-P-Int_R2
MAG_04	*Thaumarchaeota*	*Nitrososphaera*	70.8	11.04	1,219,884	718	1776	6	1.5651	BS-CT-Int_R2
MAG_05	*uc_Deltaproteobacteria*	*GR-WP33-30*	75.42	12.44	4,002,969	2515	5905	12	1.1615	BS-CT-Ext_R1
MAG_08	*Bacillaceae*	*Virgibacillus_F*	81.28	6.4	2,457,004	1229	3488	15	3.8837	RS-P-Ext_R1
MAG_10	*Flavobacteriaceae*	*Gillisia*	90.07	4.75	3,058,208	1271	4033	72	3.3439	RS-P-Ext_R1
MAG_11	*Pseudomonas*	*Pseudomonas*	97.39	4.71	4,749,133	612	4886	55	3.3354	RS-CT-Int_R1
MAG_13	*Rhizobiales*	*Methyloceanibacter*	71.18	0.9	1,553,703	289	1757	11	1.1708	BS-P-Int_R2
MAG_14	*Acidobacteria*	*OLB17*	56.83	1.71	1,904,854	464	2270	69	0.3698	BS-CT-Int_R2

^*a*^ based on Lowest Common Ancestor (LCA) as assigned by MEGAN6 of at least half of all genes in a MAG; ^*b*^ as annotated by Rapid Annotation using Subsystem Technology (RAST); ^*c*^ as calculated by average Reads Per Kilobase Million (RPKM) over all samples, considering 162 MAGs.

## Supplementary Materials

The following are available online at https://www.mdpi.com/2073-4425/10/6/424/s1. Figures S1–S5: Taxonomic profiling of the microbial communities residing in the analyzed LTE-1 soils on rank of kingdom, class, order, family and species. Relative abundances of the most abundant taxa are shown and were determined based on direct classification of reads normalized to the dataset sizes (fractions). Abbreviations stand for bulk soil (BS) or root-affected soil (RS), tillage by mould-board plough (P) or cultivator (CT), and nitrogen fertilization intensive (Int) or extensive (Ext). Figure S6: Relative abundances of genera related to plant-growth promotion (PGP), disease suppressive soils and soil health. Boxes are shaded reflecting high abundance (green), medium abundance (yellow) and low abundance (blue). Relevant genera were selected based on the following literature: [49,50,51,52,53]. The sample abbreviations are bulk soil (BS) or root-affected soil (RS), tillage by mould-board plough (P) or cultivator (CT), and nitrogen fertilization intensive (Int) or extensive (Ext). Figure S7: Relative abundances of the 35 most abundant functions according to classification of single reads regarding Enzyme Commission (EC) numbers. Relative abundances were determined based on direct classification of reads normalized to the dataset sizes (fractions). Abbreviations stand for bulk soil (BS) or root-affected soil (RS), tillage by mould-board plough (P) or cultivator (CT), and nitrogen fertilization intensive (Int) or extensive (Ext). Figure S8: Gene Ontology (GO) assignment statistics of the ontology ‘biological process’ (http://www.geneontology.org/). Figure S9: Principal coordinate analysis (PCoA) of taxa abundance data obtained from direct read classification of the soil microbial communities analyzed. Scatter plot shows principal coordinate axis 1 (PCoA1) versus principal coordinate axis 2 (PCoA2) that explain 35% and 24% of the total variance, respectively. In addition, 3092 genera were used for analysis. Cultivator-treated samples are represented by circles, mould-plough treated samples by rectangles. Abbreviations stand for bulk soil (BS) or root-affected soil (RS), tillage by mould-board plough (P) or cultivator (CT), and nitrogen fertilization intensive (Int) or extensive (Ext). Figure S10: Relative abundances of functions represented by assigned EC (Enzyme Commission) numbers related to plant-growth promotion, disease suppressive soils and soil health. Relative abundances were determined based on direct classification of reads normalized to the dataset sizes (fractions). Functions were selected based on the following literature: [79]. The sample abbreviations are bulk soil (BS) or root-affected soil (RS), tillage by mould-board plough (P) or cultivator (CT), and nitrogen fertilization intensive (Int) or extensive (Ext). Figure S11: Relative abundances of the 25 most abundant functions according to classification of single reads regarding Clusters of Orthologous Groups of proteins (COG). Relative abundances were determined based on direct classification of reads normalized to the dataset sizes (fractions). Abbreviations stand for bulk soil (BS) or root-affected soil (RS), tillage by mould-board plough (P) or cultivator (CT), and nitrogen fertilization intensive (Int) or extensive (Ext). Figure S12: Abundance of the 162 best metagenomically assembled genomes (MAGs) compiled from the LTE-1 soil microbiome and their taxonomy. The MAG abundance profile represented by RPKM (Reads Per Kilobase per Million) values is given as a heatmap; data was clustered according to correlation distance and average linkage was applied. The tree resulting from clustering was ordered (by rotation around nodes) according to higher median RPKM-values. On the right, the phylum-level taxonomic assignment according to the GTDB taxonomy of each MAG is reported. Color indicates the phylum of each MAG as assigned by GTDBtk (https://github.com/Ecogenomics/GtdbTk; *Crenarchaeota* in GTDB taxonomy corresponds to *Thaumarchaeota* in the NCBI taxonomy). In addition, 162 MAGs (>20% completeness and ≤15% contamination by means of checkM) were used for analysis. The heatmap was generated with ClustVis [124]. Abbreviations stand for bulk soil (BS) or root-affected soil (RS), tillage by mould-board plough (P) or cultivator (CT), and nitrogen fertilization intensive (Int) or extensive (Ext). Table S1: Metadata for the Long-Term-Experiment (LTE-1) field site at Bernburg-Strenzfeld (Saxony-Anhalt, Germany). Table S2: KEGG (Kyoto Encyclopedia of Genes and Genomes) pathways identified in the LTE-1 microbiome and numbers of assembled genes assigned to a certain KEGG pathway. Table S3: Key nitrogen metabolism enzymes and other marker genes detected in assembled genes of the LTE-1 microbiome and their taxonomic assignments. Table S4: Statistics of the metagenome sequencing approach for the agricultural soil microbiome of the long-term field experiment (LTE-1) located at BBG-Strenzfeld, Bernburg (Saxony-Anhalt, Germany). Table S5: Assembly of the soil metagenome originating from the long-term experimental (LTE-1) field site at BBG-Strenzfeld (Bernburg, Saxony-Anhalt, Germany), gene prediction and annotation.

## References

[1] Orgiazzi A., Ballabio C., Panagos P., Jones A., Fernández-Ugalde O. (2018). LUCAS Soil, the largest expandable soil dataset for Europe: A review. Eur. J. Soil. Sci..

[2] Blum W.E.H. (2005). Functions of Soil for Society and the Environment. Rev. Environ. Sci. Bio/Technol..

[3] Matson P.A., Parton W.J., Power A.G., Swift M.J. (1997). Agricultural intensification and ecosystem properties. Science.

[4] Tscharntke T., Clough Y., Wanger T.C., Jackson L., Motzke I., Perfecto I., Vandermeer J., Whitbread A. (2012). Global food security, biodiversity conservation and the future of agricultural intensification. Biol. Conserv..

[5] Culman S.W., Young-Mathews A., Hollander A.D., Ferris H., Sánchez-Moreno S., O’Geen A.T., Jackson L.E. (2010). Biodiversity is associated with indicators of soil ecosystem functions over a landscape gradient of agricultural intensification. Landsc. Ecol..

[6] Garbeva P., van Veen J., van Elsas J. (2004). MICROBIAL DIVERSITY IN SOIL: Selection of Microbial Populations by Plant and Soil Type and Implications for Disease Suppressiveness. Ann. Rev. Phytopathol..

[7] Torsvik V., vreås L. (2002). Microbial diversity and function in soil: From genes to ecosystems. Curr. Opin. Microbiol..

[8] Dunbar J., Barns S.M., Ticknor L.O., Kuske C.R. (2002). Empirical and theoretical bacterial diversity in four Arizona soils. Appl. Environ. Microbiol..

[9] Tringe S.G., von Mering C., Kobayashi A., Salamov A.A., Chen K., Chang H.W., Podar M., Short J.M., Mathur E.J., Detter J.C. (2005). Comparative Metagenomics of Microbial Communities. Science.

[10] Cretoiu M.S., Korthals G.W., Visser J.H.M., Van Elsas J.D. (2013). Chitin amendment increases soil suppressiveness toward plant pathogens and modulates the actinobacterial and oxalobacteraceal communities in an experimental agricultural field. Appl. Environ. Microbiol..

[11] Klein E., Katan J., Minz D., Gamliel A. (2012). Soil Suppressiveness to Fusarium Disease: Shifts in Root Microbiome Associated with Reduction of Pathogen Root Colonization. Phytopathology.

[12] Grantina-Ievina L., Nikolajeva V., Rostoks N., Skrabule I., Zarina L., Pogulis A., Ievinsh G. (2015). Impact of Green Manure and Vermicompost on Soil Suppressiveness, Soil Microbial Populations, and Plant Growth in Conditions of Organic Agriculture of Northern Temperate Climate. Organic Amendments and Soil Suppressiveness in Plant Disease Management.

[13] Feng G., Xie T., Wang X., Bai J., Tang L., Zhao H., Wei W., Wang M., Zhao Y. (2018). Metagenomic analysis of microbial community and function involved in cd-contaminated soil. BMC Microbiol..

[14] Mendes R., Kruijt M., de Bruijn I., Dekkers E., van der Voort M., Schneider J.H., Piceno Y.M., DeSantis T.Z., Andersen G.L., Bakker P.A. (2011). Deciphering the Rhizosphere Microbiome for Disease-Suppressive Bacteria. Science.

[15] Hungria M., Franchini J.C., Brandão-Junior O., Kaschuk G., Souza R.A. (2009). Soil microbial activity and crop sustainability in a long-term experiment with three soil-tillage and two crop-rotation systems. Appl. Soil Ecol..

[16] Stirling G.R., Smith M.K., Smith J.P., Stirling A.M., Hamill S.D. (2012). Organic inputs, tillage and rotation practices influence soil health and suppressiveness to soilborne pests and pathogens of ginger. Aust. Plant Pathol..

[17] Kepler R.M., Ugine T.A., Maul J.E., Cavigelli M.A., Rehner S.A. (2015). Community composition and population genetics of insect pathogenic fungi in the genus Metarhizium from soils of a long-term agricultural research system. Environ. Microbiol..

[18] Cederlund H., Wessén E., Enwall K., Jones C.M., Juhanson J., Pell M., Philippot L., Hallin S. (2014). Soil carbon quality and nitrogen fertilization structure bacterial communities with predictable responses of major bacterial phyla. Appl. Soil Ecol..

[19] Gómez Expósito R., de Bruijn I., Postma J., Raaijmakers J.M. (2017). Current Insights into the Role of Rhizosphere Bacteria in Disease Suppressive Soils. Front. Microbiol..

[20] Banik J.J., Brady S.F. (2010). Recent application of metagenomic approaches toward the discovery of antimicrobials and other bioactive small molecules. Curr. Opin. Microbiol..

[21] Thomas T., Gilbert J., Meyer F. (2014). Metagenomics: A guide from sampling to data analysis. The Role of Bioinformatics in Agriculture.

[22] Scholz M.B., Lo C.C., Chain P.S.G. (2012). Next generation sequencing and bioinformatic bottlenecks: The current state of metagenomic data analysis. Curr. Opin. Biotechnol..

[23] Culligan E.P., Sleator R.D., Marchesi J.R., Hill C. (2014). Metagenomics and novel gene discovery: Promise and potential for novel therapeutics. Virulence.

[24] Sommermann L., Geistlinger J., Wibberg D., Deubel A., Zwanzig J., Babin D., Schlüter A., Schellenberg I. (2018). Fungal community profiles in agricultural soils of a long-term field trial under different tillage, fertilization and crop rotation conditions analyzed by high-throughput ITS-amplicon sequencing. PLoS ONE.

[25] Deubel A., Hofmann B., Orzessek D. (2011). Long-term effects of tillage on stratification and plant availability of phosphate and potassium in a loess chernozem. Soil Tillage Res..

[26] Bolger A.M., Lohse M., Usadel B. (2014). Trimmomatic: A flexible trimmer for Illumina sequence data. Bioinformatics.

[27] Jaenicke S., Albaum S.P., Blumenkamp P., Linke B., Stoye J., Goesmann A. (2018). Flexible metagenome analysis using the MGX framework. Microbiome.

[28] Li D., Liu C.M., Luo R., Sadakane K., Lam T.W. (2015). MEGAHIT: An ultra-fast single-node solution for large and complex metagenomics assembly via succinct de Bruijn graph. Bioinformatics.

[29] Hyatt D., LoCascio P.F., Hauser L.J., Uberbacher E.C. (2012). Gene and translation initiation site prediction in metagenomic sequences. Bioinformatics.

[30] Buchfink B., Xie C., Huson D.H. (2015). Fast and sensitive protein alignment using DIAMOND. Nat. Methods.

[31] Huson D.H., Beier S., Flade I., Górska A., El-Hadidi M., Mitra S., Ruscheweyh H.J., Tappu R. (2016). MEGAN Community Edition - Interactive Exploration and Analysis of Large-Scale Microbiome Sequencing Data. PLoS Comput. Biol..

[32] Kang D.D., Froula J., Egan R., Wang Z. (2015). MetaBAT, an efficient tool for accurately reconstructing single genomes from complex microbial communities. PeerJ.

[33] Wu Y.W., Simmons B.A., Singer S.W. (2015). MaxBin 2.0: An automated binning algorithm to recover genomes from multiple metagenomic datasets. Bioinformatics.

[34] Sieber C.M.K., Probst A.J., Sharrar A., Thomas B.C., Hess M., Tringe S.G., Banfield J.F. (2018). Recovery of genomes from metagenomes via a dereplication, aggregation and scoring strategy. Nat. Microbiol..

[35] Jünemann S., Kleinbölting N., Jaenicke S., Henke C., Hassa J., Nelkner J., Stolze Y., Albaum S.P., Schlüter A., Goesmann A. (2017). Bioinformatics for NGS-based metagenomics and the application to biogas research. J. Biotechnol..

[36] Parks D.H., Chuvochina M., Waite D.W., Rinke C., Skarshewski A., Chaumeil P.A., Hugenholtz P. (2018). A proposal for a standardized bacterial taxonomy based on genome phylogeny. bioRxiv.

[37] Aziz R.K., Bartels D., Best A.A., DeJongh M., Disz T., Edwards R.A., Formsma K., Gerdes S., Glass E.M., Kubal M. (2008). The RAST Server: Rapid Annotations using Subsystems Technology. BMC Genomics.

[38] Zhang H., Yohe T., Huang L., Entwistle S., Wu P., Yang Z., Busk P.K., Xu Y., Yin Y. (2018). dbCAN2: A meta server for automated carbohydrate-active enzyme annotation. Nucleic Acids Res..

[39] Weber T., Blin K., Duddela S., Krug D., Kim H. (2015). antiSMASH 3.0—A comprehensive resource for the genome mining of biosynthetic gene clusters. Nucleic Acids.

[40] Blin K., Kim H.U., Medema M.H., Weber T. (2017). Recent development of antiSMASH and other computational approaches to mine secondary metabolite biosynthetic gene clusters. Brief. Bioinform..

[41] Kanehisa M., Sato Y., Morishima K. (2016). BlastKOALA and GhostKOALA: KEGG Tools for Functional Characterization of Genome and Metagenome Sequences. J. Mol. Biol..

[42] Zakrzewski M., Proietti C., Ellis J.J., Hasan S., Brion M.J., Berger B., Krause L. (2016). Calypso: A user-friendly web-server for mining and visualizing microbiome–environment interactions. Bioinformatics.

[43] Segata N., Huttenhower C. (2011). Toward an efficient method of identifying core genes for evolutionary and functional microbial phylogenies. PLoS ONE.

[44] Wood D.E., Salzberg S.L. (2014). Kraken: Ultrafast metagenomic sequence classification using exact alignments. Genome Biol..

[45] Zhalnina K., de Quadros P.D., Gano K.A., Davis-Richardson A., Fagen J.R., Brown C.T., Giongo A., Drew J.C., Sayavedra-Soto L.A., Arp D.J. (2013). Ca. Nitrososphaera and Bradyrhizobium are inversely correlated and related to agricultural practices in long-term field experiments. Front. Microbiol..

[46] Ganzert L., Lipski A., Hubberten H.W., Wagner D. (2011). The impact of different soil parameters on the community structure of dominant bacteria from nine different soils located on Livingston Island, South Shetland Archipelago, Antarctica. FEMS Microbiol. Ecol..

[47] Fierer N. (2017). Embracing the unknown: Disentangling the complexities of the soil microbiome. Nat. Rev. Microbiol..

[48] Beneduzi A., Ambrosini A., Passaglia L.M.P. (2012). Plant growth-promoting rhizobacteria (PGPR): Their potential as antagonists and biocontrol agents. Genet. Mol. Biol..

[49] Berg G. (2009). Plant-microbe interactions promoting plant growth and health: Perspectives for controlled use of microorganisms in agriculture. Appl. Microbiol. Biotechnol..

[50] Majeed A., Muhammad Z., Ahmad H. (2018). Plant growth promoting bacteria: role in soil improvement, abiotic and biotic stress management of crops. Plant Cell Rep..

[51] de Souza R., Ambrosini A., Passaglia L.M. (2015). Plant growth-promoting bacteria as inoculants in agricultural soils. Genet. Mol. Biol..

[52] Sahu P.K., Singh D.P., Prabha R., Meena K.K., Abhilash P. (2018). Connecting microbial capabilities with the soil and plant health: Options for agricultural sustainability. Ecol. Indic..

[53] Puga-Freitas R., Blouin M. (2015). A review of the effects of soil organisms on plant hormone signalling pathways. Environ. Exp. Bot..

[54] Vysloužilová B., Ertlen D., Schwartz D., Šefrna L. (2016). Chernozem. From concept to classification: A review. AUC Geogr..

[55] Hassa J., Maus I., Off S., Pühler A., Scherer P., Klocke M., Schlüter A. (2018). Metagenome, metatranscriptome, and metaproteome approaches unraveled compositions and functional relationships of microbial communities residing in biogas plants. Appl. Microbiol. Biotechnol..

[56] Howe A.C., Jansson J.K., Malfatti S.A., Tringe S.G., Tiedje J.M., Brown C.T. (2014). Tackling soil diversity with the assembly of large, complex metagenomes. Proc. Natl. Acad. Sci. USA.

[57] Lin H.H., Liao Y.C. (2016). Accurate binning of metagenomic contigs via automated clustering sequences using information of genomic signatures and marker genes. Sci. Rep..

[58] Papudeshi B., Haggerty J.M., Doane M., Morris M.M., Walsh K., Beattie D.T., Pande D., Zaeri P., Silva G.G.Z., Thompson F. (2017). Optimizing and evaluating the reconstruction of Metagenome-assembled microbial genomes. BMC Genomics.

[59] Sangwan N., Xia F., Gilbert J.A. (2016). Recovering complete and draft population genomes from metagenome datasets. Microbiome.

[60] Sczyrba A., Hofmann P., Belmann P., Koslicki D., Janssen S., Dröge J., Gregor I., Majda S., Fiedler J., Dahms E. (2017). Critical Assessment of Metagenome Interpretation—A benchmark of metagenomics software. Nat. Methods.

[61] Finn R.D., Coggill P., Eberhardt R.Y., Eddy S.R., Mistry J., Mitchell A.L., Potter S.C., Punta M., Qureshi M., Sangrador-Vegas A. (2016). The Pfam protein families database: Towards a more sustainable future. Nucleic Acids Res..

[62] Kanehisa M., Sato Y., Furumichi M., Morishima K., Tanabe M. (2019). New approach for understanding genome variations in KEGG. Nucleic Acids Res..

[63] Bulgarelli D., Schlaeppi K., Spaepen S., van Themaat E.V.L., Schulze-Lefert P. (2013). Structure and Functions of the Bacterial Microbiota of Plants. Ann. Rev. Plant Biol..

[64] Lamattina L., García-Mata C., Graziano M., Pagnussat G. (2003). NITRIC OXIDE: The Versatility of an Extensive Signal Molecule. Ann. Rev. Plant Biol..

[65] Rodríguez H., Fraga R., Gonzalez T., Bashan Y. (2006). Genetics of phosphate solubilization and its potential applications for improving plant growth-promoting bacteria. Plant Soil.

[66] Vassilev N., Martos E., Mendes G., Martos V., Vassileva M. (2013). Biochar of animal origin: A sustainable solution to the global problem of high-grade rock phosphate scarcity?. J. Sci. Food Agric..

[67] Glick B.R. (2014). Bacteria with ACC deaminase can promote plant growth and help to feed the world. Microbiol. Res..

[68] Kim Y.C., Leveau J., McSpadden Gardener B.B., Pierson E.A., Pierson L.S., Ryu C.M. (2011). The multifactorial basis for plant health promotion by plant-associated bacteria. Appl. Environ. Microbiol..

[69] Ross J.J., O’Neill D.P., Wolbang C.M., Symons G.M., Reid J.B. (2001). Auxin-Gibberellin Interactions and Their Role in Plant Growth. J. Plant Growth Regul..

[70] Fincheira P., Quiroz A. (2018). Microbial volatiles as plant growth inducers. Microbiol. Res..

[71] Kai M., Effmert U., Piechulla B. (2016). Bacterial-Plant-Interactions: Approaches to Unravel the Biological Function of Bacterial Volatiles in the Rhizosphere. Front. Microbiol..

[72] Guttenberger N., Blankenfeldt W., Breinbauer R. (2017). Recent developments in the isolation, biological function, biosynthesis, and synthesis of phenazine natural products. Bioorganic Med. Chem..

[73] Vekeman B., Kerckhof F.M., Cremers G., de Vos P., Vandamme P., Boon N., Op den Camp H.J., Heylen K. (2016). New Methyloceanibacter diversity from North Sea sediments includes methanotroph containing solely the soluble methane monooxygenase. Environ. Microbiol..

[74] Iguchi H., Yurimoto H., Sakai Y. (2015). Interactions of Methylotrophs with Plants and Other Heterotrophic Bacteria. Microorganisms.

[75] Chistoserdova L., Kalyuzhnaya M.G. (2018). Current Trends in Methylotrophy. Trends Microbiol..

[76] Kumar M., Tomar R.S., Lade H., Paul D. (2016). Methylotrophic bacteria in sustainable agriculture. World J. Microbiol. Biotechnol..

[77] Garrido-Oter R., Nakano R.T., Dombrowski N., Ma K.W., McHardy A.C., Schulze-Lefert P. (2018). Modular Traits of the Rhizobiales Root Microbiota and Their Evolutionary Relationship with Symbiotic Rhizobia. Cell Host Microbe.

[78] Costa R., Salles J.F., Berg G., Smalla K. (2006). Cultivation-independent analysis of Pseudomonas species in soil and in the rhizosphere of field-grown Verticillium dahliae host plants. Environ. Microbiol..

[79] Khan A.L., Waqas M., Kang S.M., Al-Harrasi A., Hussain J., Al-Rawahi A., Al-Khiziri S., Ullah I., Ali L., Jung H.Y. (2014). Bacterial endophyte *Sphingomonas* sp. LK11 produces gibberellins and IAA and promotes tomato plant growth. J. Microbiol..

[80] Verlinden R., Hill D., Kenward M., Williams C., Radecka I. (2007). Bacterial synthesis of biodegradable polyhydroxyalkanoates. J. Appl. Microbiol..

[81] Pankievicz V.C.S., Camilios-Neto D., Bonato P., Balsanelli E., Tadra-Sfeir M.Z., Faoro H., Chubatsu L.S., Donatti L., Wajnberg G., Passetti F. (2016). RNA-seq transcriptional profiling of Herbaspirillum seropedicae colonizing wheat (Triticum aestivum) roots. Plant Mol. Biol..

[82] Reuben S., Bhinu V.S., Swarup S. (2008). Rhizosphere Metabolomics: Methods and Applications.

[83] Behnsen J., Raffatellu M. (2016). Siderophores: More than stealing iron. mBio.

[84] Cimermancic P., Medema M., Claesen J., Kurita K., Wieland Brown L., Mavrommatis K., Pati A., Godfrey P., Koehrsen M., Clardy J. (2014). Insights into Secondary Metabolism from a Global Analysis of Prokaryotic Biosynthetic Gene Clusters. Cell.

[85] Schöner T.A., Gassel S., Osawa A., Tobias N.J., Okuno Y., Sakakibara Y., Shindo K., Sandmann G., Bode H.B. (2016). Aryl Polyenes, a Highly Abundant Class of Bacterial Natural Products, Are Functionally Related to Antioxidative Carotenoids. ChemBioChem.

[86] Bruce J.B., West S.A., Griffin A.S. (2017). Bacteriocins and the assembly of natural Pseudomonas fluorescens populations. J. Evol. Biol..

[87] Schmidt R., Cordovez V., de Boer W., Raaijmakers J., Garbeva P. (2015). Volatile affairs in microbial interactions. ISME J..

[88] John J., Saranathan R., Adigopula L.N., Thamodharan V., Singh S.P., Lakshmi T.P., CharanTej M.A., Rao R.S., Krishna R., Rao H.S.P. (2016). The quorum sensing molecule N -acyl homoserine lactone produced by Acinetobacter baumannii displays antibacterial and anticancer properties. Biofouling.

[89] Biggins J.B., Ternei M.A., Brady S.F. (2012). Malleilactone, a Polyketide Synthase-Derived Virulence Factor Encoded by the Cryptic Secondary Metabolome of Burkholderia pseudomallei Group Pathogens. J. Am. Chem. Soc..

[90] Helfrich E.J., Reiter S., Piel J. (2014). Recent advances in genome-based polyketide discovery. Curr. Opin. Biotechnol..

[91] Wanke M., Skorupinska-Tudek K., Swiezewska E. (2001). Isoprenoid biosynthesis via 1-deoxy-D-xylulose 5-phosphate/2-C-methyl-D-erythritol 4-phosphate (DOXP/MEP) pathway. Acta Biochim. Pol..

[92] Simontacchi M., García-Mata C., Bartoli C.G., Santa-María G.E., Lamattina L. (2013). Nitric oxide as a key component in hormone-regulated processes. Plant Cell Rep..

[93] Brodhagen M., Peyron M., Miles C., Inglis D.A. (2015). Biodegradable plastic agricultural mulches and key features of microbial degradation. Appl. Microbiol. Biotechnol..

[94] Mandic-Mulec I., Stefanic P., van Elsas J.D. (2015). Ecology of Bacillaceae. The Bacterial Spore: From Molecules to Systems.

[95] Mirouze N., Dubnau D. (2016). Chance and Necessity in Bacillus subtilis Development. The Bacterial Spore: From Molecules to Systems.

[96] Ransom-Jones E., McCarthy A.J., Haldenby S., Doonan J., McDonald J.E. (2017). Lignocellulose-Degrading Microbial Communities in Landfill Sites Represent a Repository of Unexplored Biomass-Degrading Diversity. mSphere.

[97] López-Mondéjar R., Zühlke D., Becher D., Riedel K., Baldrian P. (2016). Cellulose and hemicellulose decomposition by forest soil bacteria proceeds by the action of structurally variable enzymatic systems. Sci. Rep..

[98] Abe K., Nakajima M., Yamashita T., Matsunaga H., Kamisuki S., Nihira T., Takahashi Y., Sugimoto N., Miyanaga A., Nakai H. (2017). Biochemical and structural analyses of a bacterial endo-*β*-1,2-glucanase reveal a new glycoside hydrolase family. J. Biol. Chem..

[99] Shimizu H., Nakajima M., Miyanaga A., Takahashi Y., Tanaka N., Kobayashi K., Sugimoto N., Nakai H., Taguchi H. (2018). Characterization and Structural Analysis of a Novel exo -Type Enzyme Acting on *β*-1,2-Glucooligosaccharides from Parabacteroides distasonis. Biochemistry.

[100] Kwak M.J., Kong H.G., Choi K., Kwon S.K., Song J.Y., Lee J., Lee P.A., Choi S.Y., Seo M., Lee H.J. (2018). Rhizosphere microbiome structure alters to enable wilt resistance in tomato. Nat. Biotechnol..

[101] Piccoli P., Bottini R. (2013). Terpene Production by Bacteria and its Involvement in Plant Growth Promotion, Stress Alleviation, and Yield Increase. Molecular Microbial Ecology of the Rhizosphere.

[102] Yu D., Xu F., Zeng J., Zhan J. (2012). Type III polyketide synthases in natural product biosynthesis. IUBMB Life.

[103] Katsuyama Y., Ohnishi Y. (2012). Type III Polyketide Synthases in Microorganisms. Methods Enzymol..

[104] Kielak A.M., Barreto C.C., Kowalchuk G.A., van Veen J.A., Kuramae E.E. (2016). The Ecology of Acidobacteria: Moving beyond Genes and Genomes. Front. Microbiol..

[105] Eichorst S.A., Trojan D., Roux S., Herbold C., Rattei T., Woebken D. (2018). Genomic insights into the Acidobacteria reveal strategies for their success in terrestrial environments. Environ. Microbiol..

[106] Kielak A.M., Cipriano M.A.P., Kuramae E.E. (2016). Acidobacteria strains from subdivision 1 act as plant growth-promoting bacteria. Arch. Microbiol..

[107] Pester M., Schleper C., Wagner M. (2011). The Thaumarchaeota: An emerging view of their phylogeny and ecophysiology. Curr. Opin. Microbiol..

[108] Tourna M., Stieglmeier M., Spang A., Konneke M., Schintlmeister A., Urich T., Engel M., Schloter M., Wagner M., Richter A. (2011). Nitrososphaera viennensis, an ammonia oxidizing archaeon from soil. Proc. Natl. Acad. Sci. USA.

[109] Offre P., Nicol G.W., Prosser J.I. (2011). Community profiling and quantification of putative autotrophic thaumarchaeal communities in environmental samples. Environ. Microbiol. Rep..

[110] Nicol G.W., Schleper C. (2006). Ammonia-oxidising Crenarchaeota: Important players in the nitrogen cycle?. Trends Microbiol..

[111] Könneke M., Schubert D.M., Brown P.C., Hügler M., Standfest S., Schwander T., Schada von Borzyskowski L., Erb T.J., Stahl D.A., Berg I.A. (2014). Ammonia-oxidizing archaea use the most energy-efficient aerobic pathway for CO2 fixation. Proc. Natl. Acad. Sci. USA.

[112] Berg I.A., Kockelkorn D., Buckel W., Fuchs G. (2007). A 3-hydroxypropionate/4-hydroxybutyrate autotrophic carbon dioxide assimilation pathway in archaea. Science.

[113] Cibis K.G., Gneipel A., König H. (2016). Isolation of acetic, propionic and butyric acid-forming bacteria from biogas plants. J. Biotechnol..

[114] Müller H., Berg C., Landa B.B., Auerbach A., Moissl-Eichinger C., Berg G. (2015). Plant genotype-specific archaeal and bacterial endophytes but similar Bacillus antagonists colonize Mediterranean olive trees. Front. Microbiol..

[115] Hedden P., Thomas S.G. (2012). Gibberellin biosynthesis and its regulation. Biochem. J..

[116] Cuskin F., Baslé A., Ladevèze S., Day A.M., Gilbert H.J., Davies G.J., Potocki-Véronèse G., Lowe E.C. (2015). The GH130 Family of Mannoside Phosphorylases Contains Glycoside Hydrolases That Target *β*-1,2-Mannosidic Linkages in Candida Mannan. J. Biol. Chem..

[117] Hong J.K., Cho J.C. (2015). Environmental Variables Shaping the Ecological Niche of Thaumarchaeota in Soil: Direct and Indirect Causal Effects. PLoS ONE.

[118] Kielak A., Pijl A.S., van Veen J.A., Kowalchuk G.A. (2009). Phylogenetic diversity of Acidobacteria in a former agricultural soil. ISME J..

[119] Schreiter S., Ding G.C., Heuer H., Neumann G., Sandmann M., Grosch R., Kropf S., Smalla K. (2014). Effect of the soil type on the microbiome in the rhizosphere of field-grown lettuce. Front. Microbiol..

[120] Feng L., Chen K., Han D., Zhao J., Lu Y., Yang G., Mu J., Zhao X. (2017). Comparison of nitrogen removal and microbial properties in solid-phase denitrification systems for water purification with various pretreated lignocellulosic carriers. Bioresour. Technol..

[121] Gupta R.S., Son J., Oren A. (2018). A phylogenomic and molecular markers based taxonomic framework for members of the order Entomoplasmatales: proposal for an emended order Mycoplasmatales containing the family Spiroplasmataceae and emended family Mycoplasmataceae comprised of six genera. Antonie van Leeuwenhoek.

[122] Torres-Cortés G., Ghignone S., Bonfante P., Schüßler A. (2015). Mosaic genome of endobacteria in arbuscular mycorrhizal fungi: Transkingdom gene transfer in an ancient mycoplasma-fungus association. Proc. Natl. Acad. Sci. USA.

[123] Treseder K.K., Allen E.B., Egerton-Warburton L.M., Hart M.M., Klironomos J.N., Maherali H., Tedersoo L. (2018). Arbuscular mycorrhizal fungi as mediators of ecosystem responses to nitrogen deposition: A trait-based predictive framework. J. Ecol..

[124] Metsalu T., Vilo J. (2015). ClustVis: A web tool for visualizing clustering of multivariate data using Principal Component Analysis and heatmap. Nucleic Acids Res..

