# Supplementary figures and images for: Effect of Long-Term Farming Practices on Agricultural Soil Microbiome Members Represented by Metagenomically Assembled Genomes (MAGs) and Their Predicted Plant-Beneficial Genes
Source: Genes (Basel). 2019 Jun 3;10(6):424. doi: 10.3390/genes10060424 (PMC6627896; doi:10.3390/genes10060424)

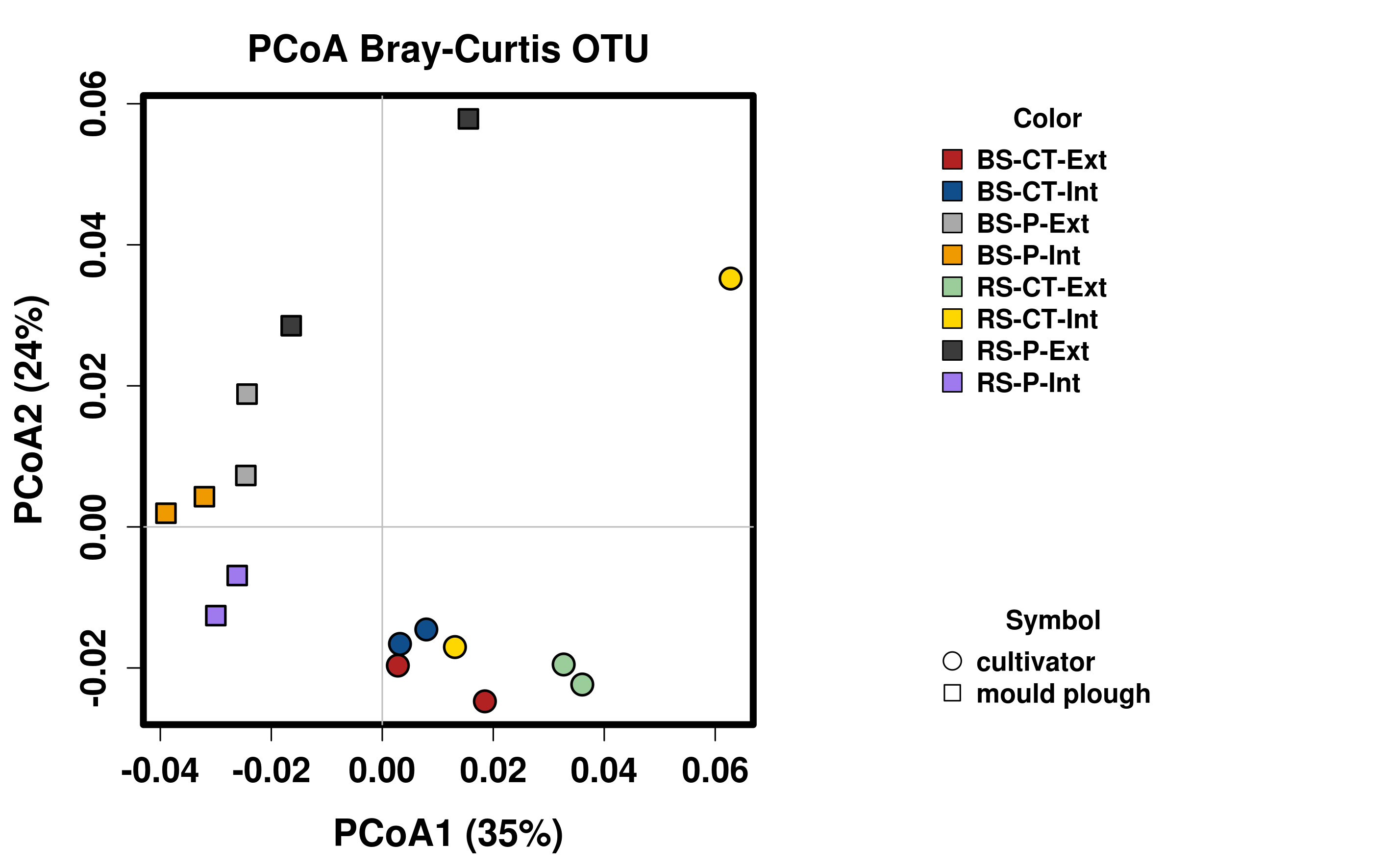

Supplement: Supplementary file 1 [file genes-10-00424-s001.zip › Supplement/S09_calypso-3137225844448881680.multivar_Genus.png]

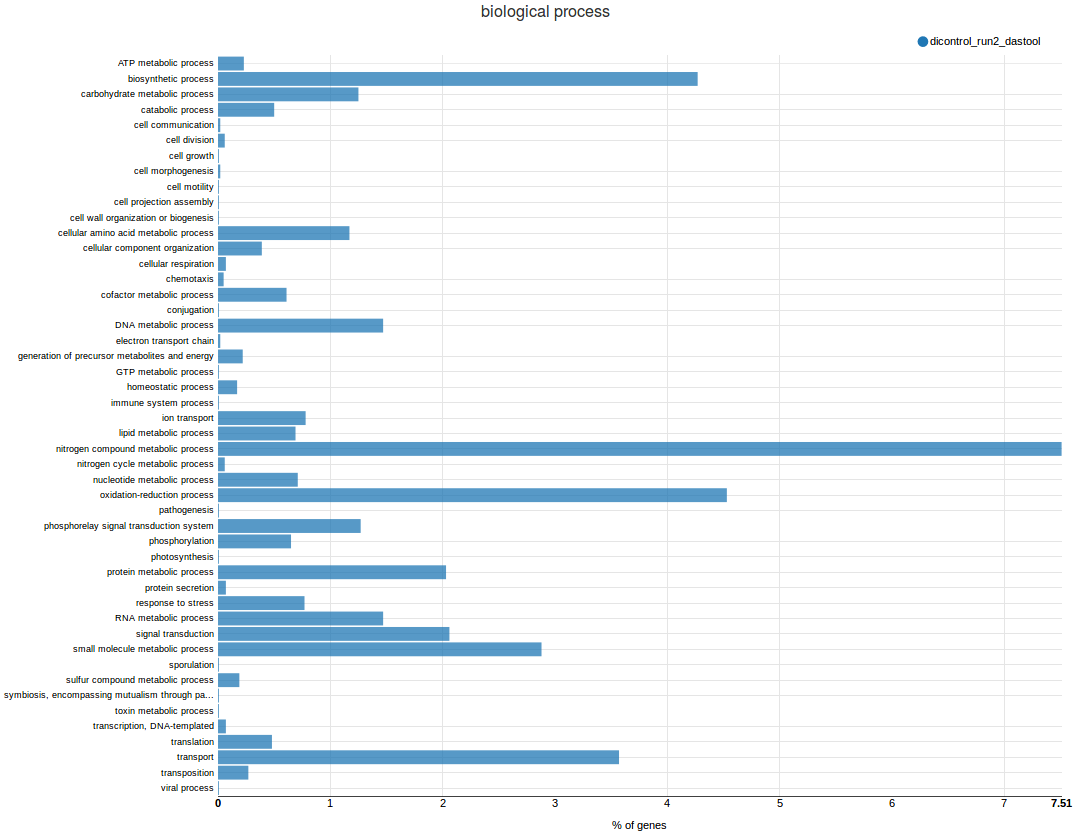

Supplement: Supplementary file 1 [file genes-10-00424-s001.zip › Supplement/S08_GO_biologicalprocess.png]

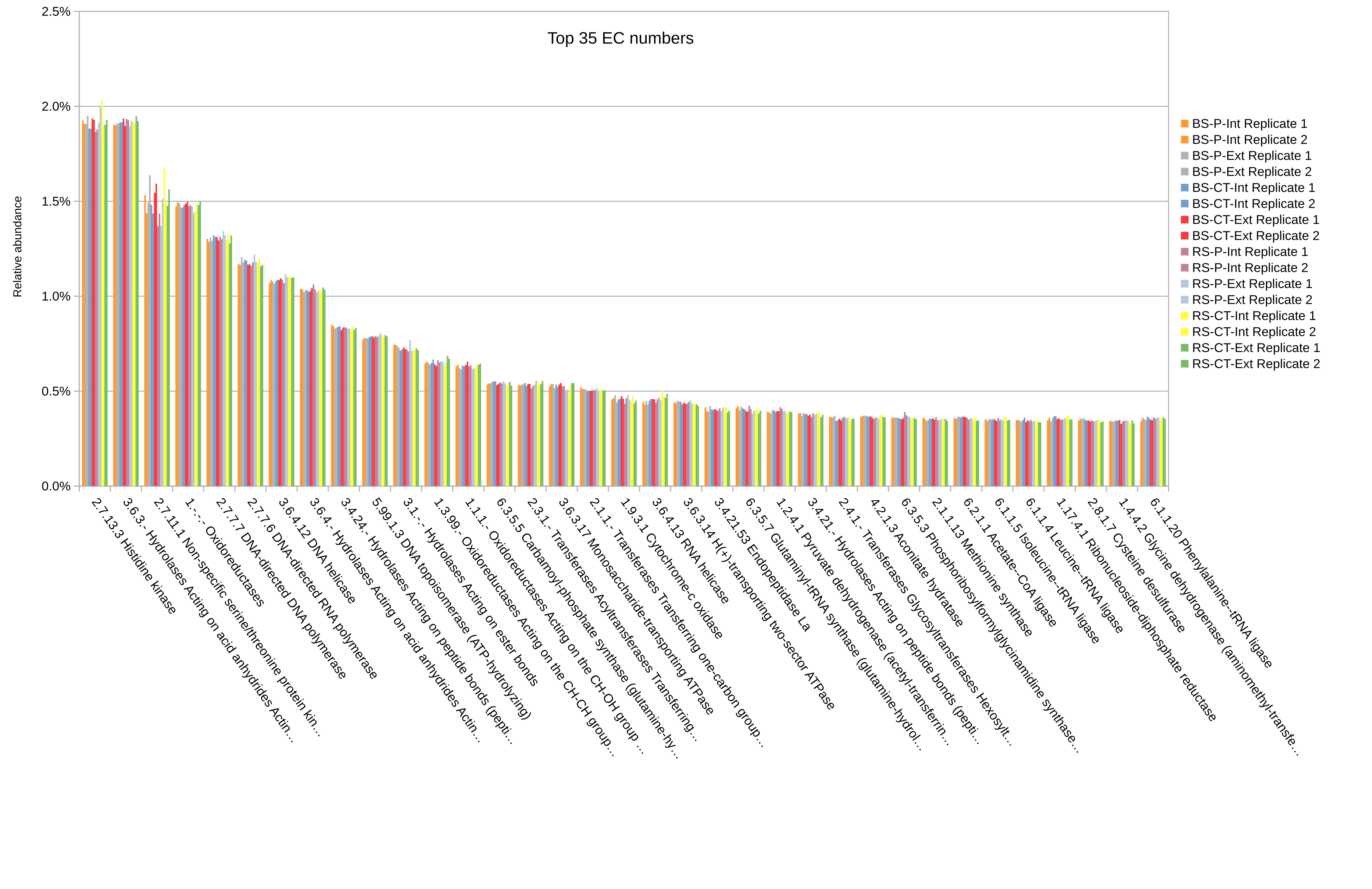

Supplement: Supplementary file 1 [file genes-10-00424-s001.zip › Supplement/S07_Top35_ECs.png]

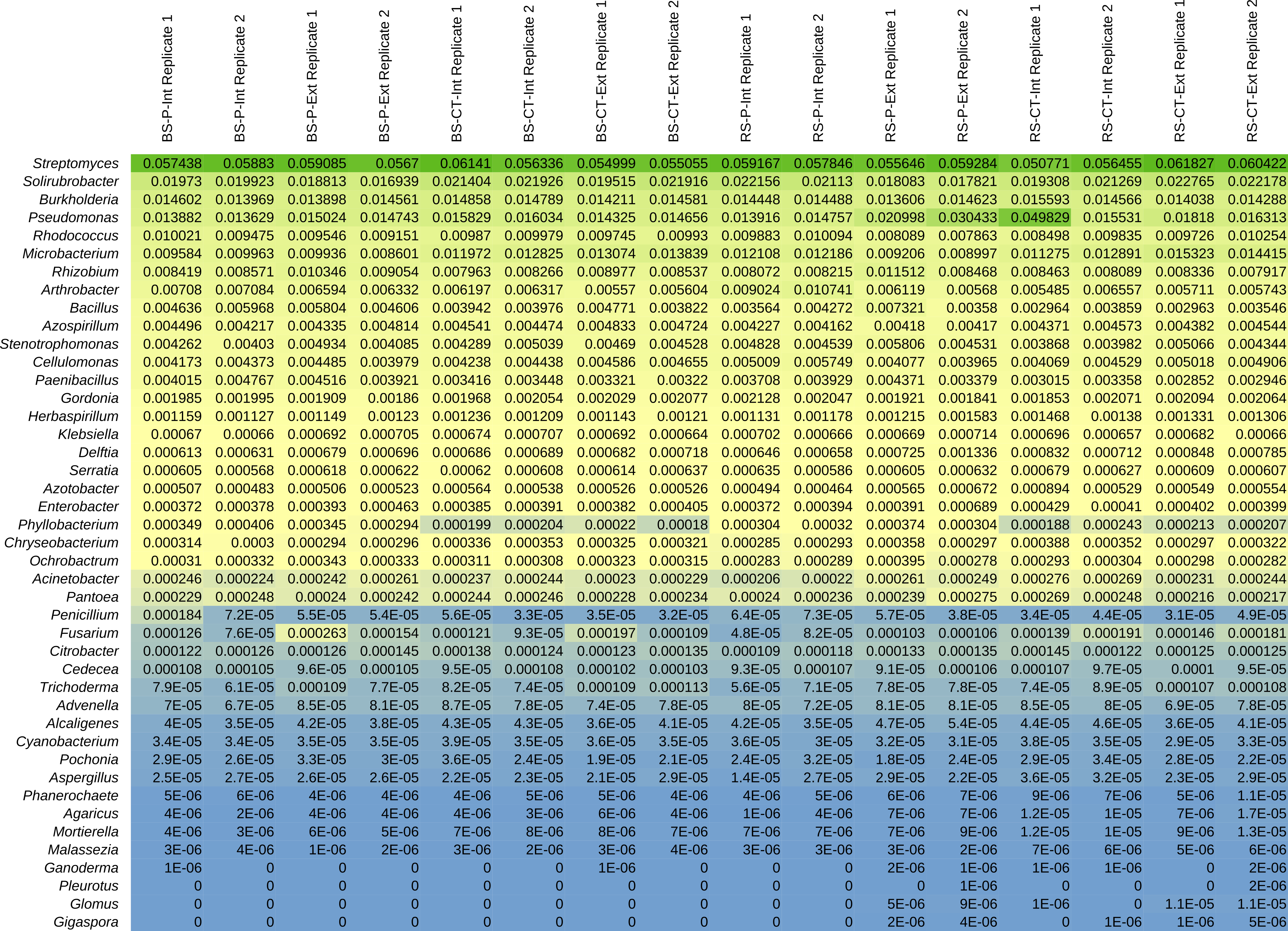

Supplement: Supplementary file 1 [file genes-10-00424-s001.zip › Supplement/S06_Genera_of_Interest.png]

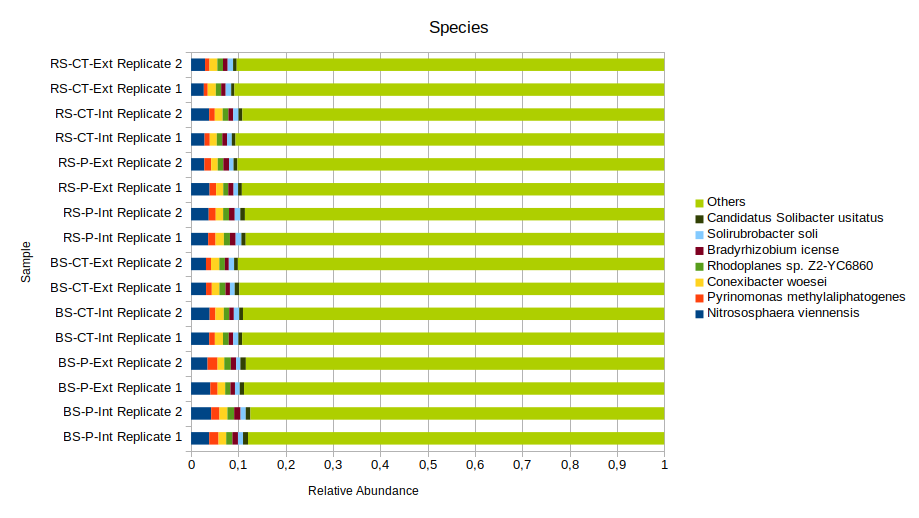

Supplement: Supplementary file 1 [file genes-10-00424-s001.zip › Supplement/S05_Species.png]

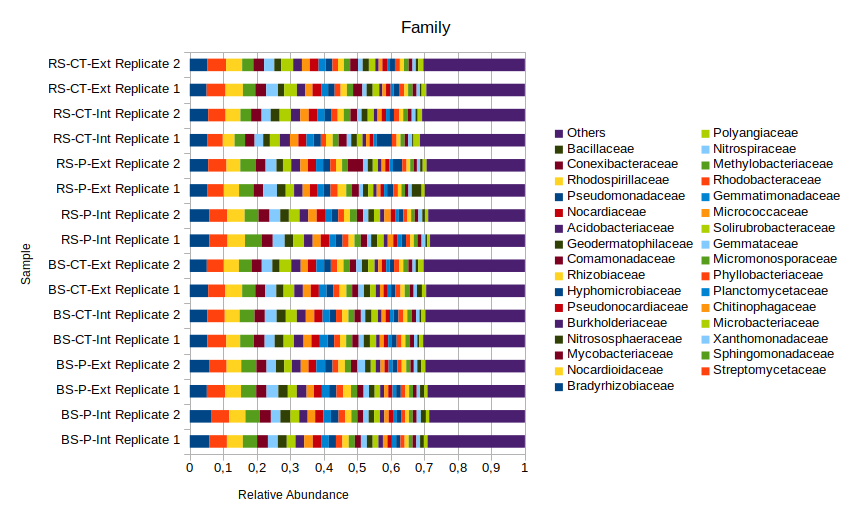

Supplement: Supplementary file 1 [file genes-10-00424-s001.zip › Supplement/S04_Family.png]

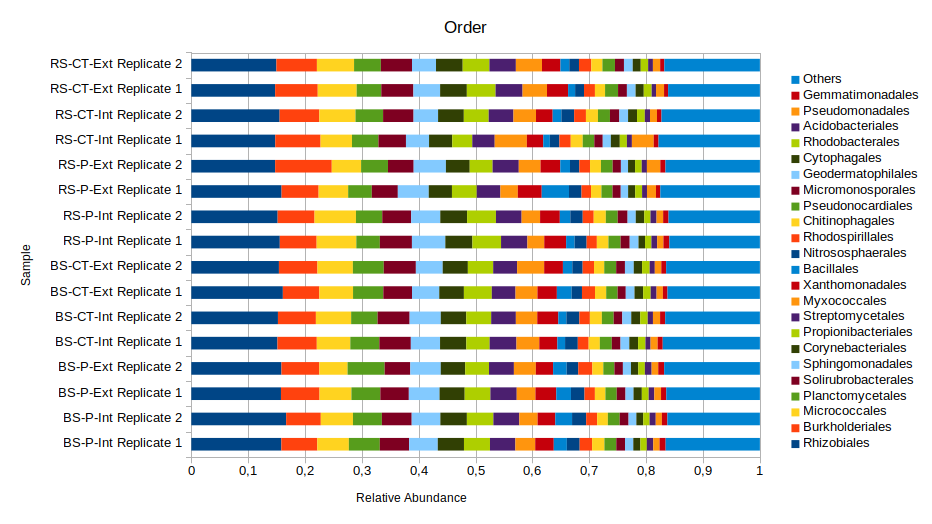

Supplement: Supplementary file 1 [file genes-10-00424-s001.zip › Supplement/S03_Order.png]

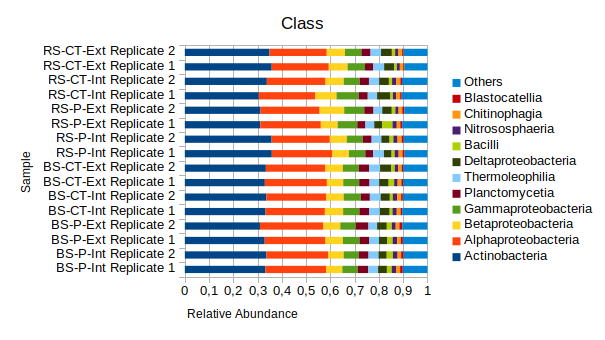

Supplement: Supplementary file 1 [file genes-10-00424-s001.zip › Supplement/S02_Class.png]

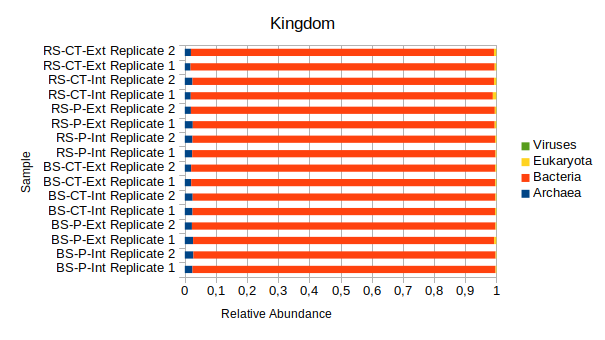

Supplement: Supplementary file 1 [file genes-10-00424-s001.zip › Supplement/S01_Kingdom.png]

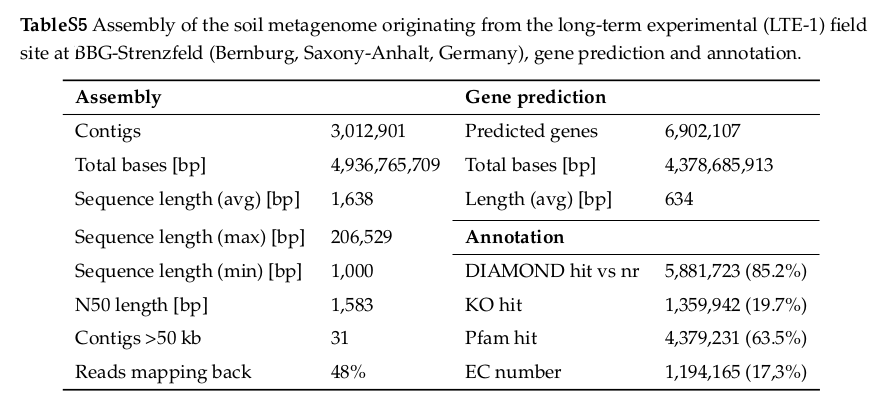

Supplement: Supplementary file 1 [file genes-10-00424-s001.zip › Supplement/TS5_AssemblyStatistics.png]

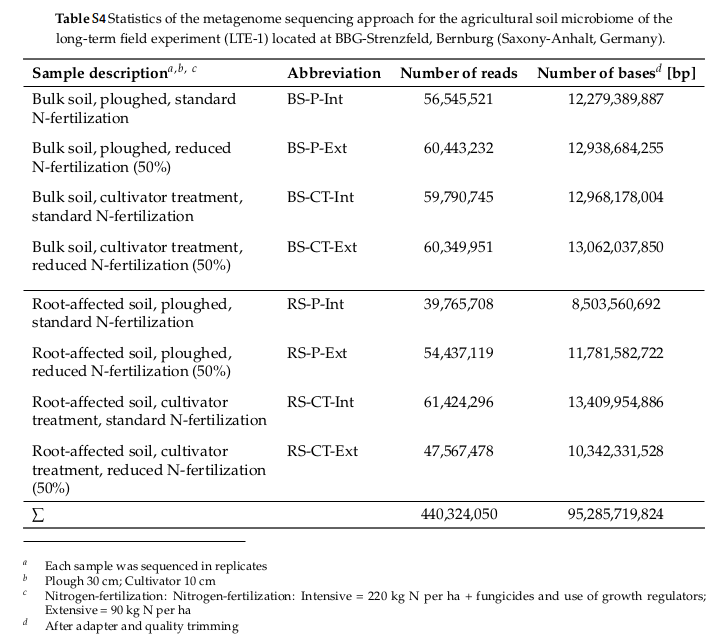

Supplement: Supplementary file 1 [file genes-10-00424-s001.zip › Supplement/TS4_SequencingStatistics.png]
