# Supplementary material for: Effect of Long-Term Farming Practices on Agricultural Soil Microbiome Members Represented by Metagenomically Assembled Genomes (MAGs) and Their Predicted Plant-Beneficial Genes
Source: Genes (Basel). 2019 Jun 3;10(6):424. doi: 10.3390/genes10060424 (PMC6627896; doi:10.3390/genes10060424)
Supplement: Supplementary file 6 [file genes-10-00424-s001.zip › Supplement/TS3_Table_assembled_marker_genes_20181106.odt]

Table S3: Identification of marker genes encoding enzymes of interest in the assembled LTE-1 soil metagenome dataset

| Taxon (phylum level) | Abundancea (percentage) | EC 3.5.1.5 Urease (ureC) b, c | EC 1.7.2.4 Nitrous-oxide reductase (nosZ) b, c | EC 1.7.2.5 Nitric oxide reductase subunit B (norB) b, c | EC 1.14.99.39 Ammonia monooxygenase; (amo) b, c | EC 1.7.2.6 Hydroxylamine dehydrogenase (hao) b, c | EC 6.3.1.2 Glutamine synthetase (glnA) b, c | EC 1.7.2.1 Nitrite reductase (NO-forming) (nirK) b, c | EC 1.7.99.1 Hydroxylamine reductaseb, c |
| --- | --- | --- | --- | --- | --- | --- | --- | --- | --- |
| Actinobacteria | 22.9 | 274 (24.2%) |  | 94 (21.7%) | 3 (12.5%) |  | 1575 (36.8%) | 71 (11.2%) |  |
| Proteobacteria | 21.9 | 434 (38.4%) | 41 (17.1%) | 173 (39.9%) | 1 (4.2%) | 1 (20%) | 1070 (25%) | 259 (40.8%) | 28 (80%) |
| Acidobacteria | 9.2 | 17 (1.5%) | 10 (4.2%) | 13 (3.0%) |  |  | 262 (6.1%) | 2 (0.3%) |  |
| Bacteroidetes | 2.9 | 10 (0.9%) | 69 (28.8%) | 13 (3.0%) |  |  | 109 (2.5%) | 24 (3.8%) |  |
| Chloroflexi | 2.9 | 30 (2.7%) | 2 (0.8%) |  |  |  | 186 (4.3%) | 41 (6.5%) |  |
| Gemmatimonadetes | 1.7 |  | 9 (3.8%) |  |  |  | 80 (1.9%) | 1 (0.16%) |  |
| Planctomycetes | 1.5 |  |  | 5 (1.2%) |  |  | 24 (0.6%) |  |  |
| Firmicutes | 0.8 | 2 (0.2%) |  | 2 (0.5%) |  |  | 23 (0.5%) |  |  |
| Verrucomicrobia | 0.6 | 7 (0.6%) | 10 (4.2%) |  |  |  | 28 (0.65%) | 6 (0.9%) |  |
| Cyanobacteria | 0.5 | 5 (0.4%) |  | 1 (0.23%) |  |  | 9 (0.2%) |  |  |
| Nitrospirae | 0.4 | 9 (0.8%) |  |  |  |  | 6 (0.14%) | 9 (1.4%) |  |
| Armatimonadetes | 0.2 |  |  |  |  |  | 1 (0.02%) |  |  |
| Tenericutes | 0.1 |  |  |  |  |  |  |  |  |
| Deinococcus-Thermus | 0.06 | 1 (0.1%) |  |  |  |  | 1 (0.02%) |  |  |
| Chlorobi | 0.02 |  |  |  |  |  |  |  |  |
| Thaumarchaeota | 1.2 | 80 (7.1%) |  |  |  |  | 91 (2.1%) | 16 (2.5%) |  |
| Euryarchaeota | 0.1 |  | 2 (0.8%) |  |  |  |  |  |  |
| Crenarchaeota | 0.03 |  | 3 (1.3%) |  |  |  |  | 12 (1.9%) |  |
| Otherk | 33 | 262 (23.2%) | 94 (39.2%) | 133 (30.6%) | 20 (83.3%) | 4 (80%) | 817 (19.1%) | 194 (30.6%) | 7 (20%) |
| Total no. of GOIsm |  | 1131 | 240 | 434 | 24 | 5 | 4282 | 635 | 35 |

Table S3: Identification of marker genes encoding enzymes of interest in the assembled LTE-1 soil metagenome dataset (cont.)

| Taxon (phylum level) | Abundancea (percentage) | EC 3.1.3.2 Acid phosphataseb, d | EC 3.1.3.8 3-Phytaseb, d | EC 1.1.5.2 Glucose 1-dehydrogenase (PQQ, quinone) b, d | EC 1.3.3.11 Pyrroloquinoline-quinone synthase  (pqqC) b, d | EC 3.5.99.7 1-Aminocyclo-propane-1-carboxylate deaminase (acdS) b, e | EC 3.5.1.4 Amidaseb, f | EC 4.1.1.74 Indolepyruvate decarboxylaseb, f | EC 1.2.1.3 Aldehyde dehydrogenase (NAD+)b, f |
| --- | --- | --- | --- | --- | --- | --- | --- | --- | --- |
| Actinobacteria | 22.9 | 6 (4.3%) | 40 (15.1%) | 2 (0.07%) | 124 (29.7%) | 135 (69.2%) | 1114 (31.2%) | 17 (8.7%) | 837 (28.3%) |
| Proteobacteria | 21.9 | 77 (55%) | 100 (37.7%) | 264 (9.4%) | 123 (29.5%) | 44 (22.6%) | 740 (20.7%) | 67 (34.2%) | 767 (25.9%) |
| Acidobacteria | 9.2 | 2 (1.4%) | 20 (7.5%) | 1573 (55.9%) | 2 (0.5%) |  | 308 (8.6%) |  | 314 (10.6%) |
| Bacteroidetes | 2.9 | 23 (16.4%) | 5 (1.9%) | 193 (6.9%) |  | 7 (3.6%) | 13 (0.4%) | 1 (0.5%) | 119 (4.0%) |
| Chloroflexi | 2.9 |  |  |  |  |  | 24 (0.7%) |  | 90 (3.0%) |
| Gemmatimonadetes | 1.7 | 1 (0.7%) |  | 116 (4.1%) |  |  | 60 (1.7%) |  | 65 (2.2%) |
| Planctomycetes | 1.5 |  | 2 (0.8%) | 7 (0.2%) |  |  | 1 (0.03%) | 30 (15.3%) | 22 (0.7%) |
| Firmicutes | 0.8 | 1 (0.7%) |  | 3 (0.1%) |  |  | 19 (0.5%) |  | 41 (1.4%) |
| Verrucomicrobia | 0.6 |  |  | 19 (0.7%) | 2 (0.5%) |  | 10 (0.3%) | 1 (0.5%) | 6 (0.2%) |
| Cyanobacteria | 0.5 | 1 (0.7%) | 18 (6.8%) |  | 2 (0.5%) |  | 9 (0.3%) |  | 8 (0.3%) |
| Nitrospirae | 0.4 | 3 (2.1%) | 1 (0.4%) |  | 11 (2.6%) |  |  | 12 (6.1%) | 6 (0.2%) |
| Armatimonadetes | 0.2 |  |  |  | 6 (1.4%) |  |  |  | 6 (0.2%) |
| Tenericutes | 0.1 |  |  |  |  |  |  |  |  |
| Deinococcus-Thermus | 0.06 |  |  |  |  |  |  |  | 4 (0.1%) |
| Chlorobi | 0.02 |  |  |  | 1 (0.24%) |  |  |  | 1 (0.03%) |
| Thaumarchaeota | 1.2 |  |  |  | 28 (6.7%) |  |  |  |  |
| Euryarchaeota | 0.1 |  |  |  |  |  | 2 (0.06%) |  |  |
| Crenarchaeota | 0.03 |  |  |  |  |  |  |  |  |
| Otherk | 33 | 26 (18.6%) | 79 (29.8%) | 637 (22.6%) | 118 (28.3%) | 9 (4.6%) | 1275 (35.7%) | 68 (34.7%) | 675 (22.8%) |
| Total no. of GOIsm |  | 140 | 265 | 2814 | 417 | 195 | 3575 | 196 | 2961 |

Table S3: Identification of marker genes encoding enzymes of interest in the assembled LTE-1 soil metagenome dataset (cont.)

| Taxon | Abundancea (percentage) | EC 2.2.1.6 Acetolactate synthaseb, g | EC 4.1.1.5 Acetolactate decarboxylaseb, g | EC 1.1.1.76 (S,S)-butanediol dehydrogenaseb, g | EC 1.1.1.4 (R,R)-butanediol dehydrogenaseb, g | EC 5.3.3.17 trans-2,3-dihydro-3-hydroxyanthranilate isomeraseb, h | EC 4.1.1.39 Ribulose-bisphosphate carboxylase | EC 3.2.1.14 Chitinaseb, i | EC 3.2.1.4 Cellulaseb, j |
| --- | --- | --- | --- | --- | --- | --- | --- | --- | --- |
| Actinobacteria | 22.9 | 1318 (23.5%) | 38 (44.7%) | 47 (24.2%) | 89 (60.5%) | 170 (29.1%) | 53 (19.6%) | 86 (16.3%) | 370 (38.1%) |
| Proteobacteria | 21.9 | 2175 (38.7%) | 2 (2.4%) | 37 (19.1%) | 19 (12.9%) | 137 (23.5%) | 126 (46.7%) | 102 (19.4%) | 115 (11.9%) |
| Acidobacteria | 9.2 | 400 (7.1%) |  | 9 (4.6%) |  | 111 (19%) |  | 30 (5.7%) | 2 (0.2%) |
| Bacteroidetes | 2.9 | 72 (1.3%) | 4 (4.7%) | 1 (0.5%) |  |  | 11 (4.1%) | 42 (8.0%) | 55 (5.7%) |
| Chloroflexi | 2.9 | 310 (5.5%) |  | 2 (1%) | 1 (0.7%) | 4 (0.7%) |  | 7 (1.3%) | 68 (7.0%) |
| Gemmatimonadetes | 1.7 | 36 (0.6%) |  |  |  | 5 (0.86%) |  | 4 (0.8%) | 57 (5.9%) |
| Planctomycetes | 1.5 | 69 (1.2%) | 4 (4.7%) |  | 3 (2.0%) |  | 3 (1.1%) | 7 (1.3%) | 31 (3.2%) |
| Firmicutes | 0.8 | 45 (0.8%) |  |  | 3 (2.0%) |  |  | 14 (2.7%) | 27 (2.8%) |
| Verrucomicrobia | 0.6 | 24 (0.4%) |  |  |  | 2 (0.34%) |  | 6 (1.1%) | 21 (2.2%) |
| Cyanobacteria | 0.5 | 9 (0.2%) | 1 (1.2%) |  |  |  | 1 (0.4%) | 2 (0.4%) | 1 (01%) |
| Nitrospirae | 0.4 | 27 (0.5%) |  |  |  | 12 (2.1%) | 1 (0.4%) | 3 (0.6%) |  |
| Armatimonadetes | 0.2 |  |  | 6 (3.1%) |  |  |  |  |  |
| Tenericutes | 0.1 |  |  |  |  |  |  |  |  |
| Deinococcus-Thermus | 0.06 |  |  |  |  |  |  | 1 (0.2%) | 1 (01%) |
| Chlorobi | 0.02 |  |  |  |  |  |  |  | 1 (01%) |
| Thaumarchaeota | 1.2 | 86 (1.5%) |  |  |  |  |  | 1 (0.2%) |  |
| Euryarchaeota | 0.1 |  |  |  |  | 2 (0.34%) |  |  |  |
| Crenarchaeota | 0.03 |  |  |  |  |  |  |  |  |
| Otherk | 33 | 1049 (18.7%) | 36 (42.4%) | 92 (47.4%) | 32 (21.8%) | 141 (24.1%) | 75 (27.8%) | 222 (42.1%) | 221 (22.8%) |
| Total no. of GOIsm |  | 5620 | 85 | 194 | 147 | 584 | 270 | 527 | 970 |

a Percentage of genes assigned to the denoted taxon (phylum); 100% corresponds to 6,902,107 genes.

b Percentage of the genes of interest (GOI) assigned to a particular taxon (phylum); 100% corresponds to all genes encoding a specific enzyme classified by its EC number.

c Enzyme of the functional context ‘nitrogen metabolism’

d Enzyme of the functional context ‘phosphorus solubilization’

e Enzyme of the functional context ‘interference with ethylene phytohormone level’

f Enzyme of the functional context ‘indole acetic acid (IAA) synthesis’

g Enzyme of the functional context ‘volatile organic compound (VOC) synthesis’

h Enzyme of the functional context ‘phenazine synthesis’

i Enzyme of the functional context ‘carbon dioxide (CO2) fixation’

j Enzyme of the functional context ‘carbohydrate hydrolysis’

k Percentage of genes or GOIs (genes of interest) assigned to unclassified bacteria, unclassified archaea, unclassified-unknown taxa, candidate taxa or Eukaryota 

m GOIs (genes of interest) assigned to unclassified bacteria, unclassified archaea, unclassified-unknown taxa, candidate taxa or Eukaryota were not differentiated in this Table
