# Supplementary material for: Effect of Long-Term Farming Practices on Agricultural Soil Microbiome Members Represented by Metagenomically Assembled Genomes (MAGs) and Their Predicted Plant-Beneficial Genes
Source: Genes (Basel). 2019 Jun 3;10(6):424. doi: 10.3390/genes10060424 (PMC6627896; doi:10.3390/genes10060424)
Supplement: Supplementary file 6 [file genes-10-00424-s001.zip › Supplement/TS1_TableS1_Metadata_LTE1_20190208.odt]

Table S1: Metadata for the Long-Term-Experiment (LTE-1) field site at Bernburg-Strenzfeld (Saxony-Anhalt, Germany)

| Location | Bernburg-Strenzfeld (LTE-1 field site), Saxony-Anhalt, Germany |
| --- | --- |
| Latitude/Longitude | 51.82 ° (N), 11.70 ° (E) |
| Altitude | 80 m (above sea level) |
| Environment | Agricultural farmland |
| Cultivated crop | Winter wheat cultivar ‘Pamir’ |
| Soil type | Loess chernozem over limestone |
| Soil texture | 22% clay, 70% silt, 8% sand |
| Horizon (sampled) | Ap |
| pH | 7.0 – 7.4 |
| P supply | 45 – 70 mg kg-1 |
| K supply | 130 – 185 mg kg-1 |
| Average annual temperature (1980 – 2010) | 9.7 °C |
| Average annual precipitation | 511 mm |
| Crop rotation | Grain maize (Zea mays), winter wheat (Triticum aestivum), winter barley (Hordeum vulgare), winter rapeseed (Brassica napus ssp. Napus), winter wheat |
| Treatment: N-fertilization | 220 kg per ha as ammonium sulfate and calcium ammonium nitrate (intensive - Int) or 90 kg per ha as ammonium sulfate and calcium ammonium nitrate (extensive – Ext) |
| Treatment: Tillage | Ploughed using a mould-board plough (P), carrier board with combined alternating ploughshares, ploughing depth 30 cm, incl. soil inversion or treated with cultivator (CT), 10 cm flat non-inverting soil loosening |
| History | Long-term field trial started in 1992 |
| Collection date | 30. July 2015 |
| Sampling depth | 0 – 25 cm (soil corer) |
| Physicochemical soil properties | Deubel et al., 2011 |
| Basic soil parameters | Sommermann et al., 2018; Table S3 |
| Winter wheat yields of the LTE-1 field plots | Described by Sommermann et al., 2018 |

Data taken from Sommermann et al., 2018, PLOS ONE, 13 (4):e0195345. doi: 10.1371/journal.pone.0195345

Deubel A, Hofmann B, Orzessek D. Long-term effects of tillage on stratification and plant availability of phosphate and potassium in a loess chernozem. Soil Till Res 2011;117:85–92.
